# Origin and adaptation of green‐sensitive (RH2) pigments in vertebrates

**DOI:** 10.1002/2211-5463.12843

**Published:** 2020-04-11

**Authors:** Shozo Yokoyama, Huiyong Jia

**Affiliations:** ^1^ Department of Biology Emory University Atlanta GA USA; ^2^ Willamette View Portland OR USA

**Keywords:** adaptive evolution, ancestral pigment reconstruction, gene duplication, RH2 opsin, spectral tuning

## Abstract

One of the critical times for the survival of animals is twilight where the most abundant visible lights are between 400 and 550 nanometres (nm). Green‐sensitive RH2 pigments help nonmammalian vertebrate species to better discriminate wavelengths in this blue‐green region. Here, evaluation of the wavelengths of maximal absorption (λ_max_s) of genetically engineered RH2 pigments representing 13 critical stages of vertebrate evolution revealed that the RH2 pigment of the most recent common ancestor of vertebrates had a λ_max_ of 503 nm, while the 12 ancestral pigments exhibited an expanded range in λ_max_s between 474 and 524 nm, and present‐day RH2 pigments have further expanded the range to ~ 450–530 nm. During vertebrate evolution, eight out of the 16 significant λ_max_ shifts (or |Δλ_max_| ≥ 10 nm) of RH2 pigments identified were fully explained by the repeated mutations E122Q (twice), Q122E (thrice) and M207L (twice), and A292S (once). Our data indicated that the highly variable λ_max_s of teleost RH2 pigments arose from gene duplications followed by accelerated amino acid substitution.

## Graphical abstract

Thirteen genetically engineered ancestral RH2 pigments show that the pigment of the vertebrate ancestor absorbed the wavelength maximally (λ_max_) at 503 nm. The absorption capability was expanded to 450–530 nm among its descendants. Eight out of 16 significant λ_max_ shifts during vertebrate evolution are explained by several mutations, but the mutations causing the other functional changes remain to be identified.

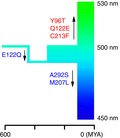

**Abbreviations**
λ_max_: the wavelength of maximal absorption
C terminus: carboxyl‐terminus
JTT: Jones–Taylor–Thornton
M/LWS: middle‐ and long‐wavelength‐sensitive
N terminus: amino‐terminus
PAML: phylogenetic analyses by maximum likelihood
RH1: rhodopsin
RH2: rhodopsin‐like
SWS1: shortwave length‐sensitive type 1
SWS2: SWS type 2
WAG: Whelan and Goldman

Vision is initiated by the absorption of lights by visual pigments, each of which consists of an opsin, encoded by an opsin gene, and the retinal (11‐*cis*‐retinal or 11‐*cis*‐3, 4‐dehydroretinal) derived from diet [1, 2, 3, 4]. Prior to the appearance of the vertebrate ancestor, five groups of visual pigments already existed: rhodopsin (or RH1), rhodopsin‐like (or RH2), shortwave length‐sensitive type 1 (SWS1), SWS type 2 (SWS2) and middle‐ and long‐wavelength‐sensitive (M/LWS) pigments that absorb light maximally (λ_max_) at 480–510, 450–530, 360–440, 400–450 and 510–570 nm, respectively [5, 6, 7].

One of the critical times for the survival of organisms is twilight [8] where prevalent wavelengths are between 400 and 550 nanometres (nm) [9, 10]. As lights pass through to deeper depth in the clear oceans and lakes, the amount of light is reduced and short and long wavelengths are absorbed by water and the remaining lights also become limited to about 480 nm [11]. Visual acuity of many species in these dim‐light environments has been improved by gene duplications within RH1 [12], RH2 [13, 14, 15, 16, 17] and SWS2 pigment lineages [14, 15, 18, 19, 20, 21, 22, 23, 24, 25, 26].

The molecular bases of the λ_max_ shift (or spectral tuning) in visual pigments have been studied mostly by introducing various mutations into various present‐day pigments. This approach is based on two implicit assumptions: (a) identical amino acid changes in different pigments shift the λ_max_ by the same magnitude and (b) forward and reverse mutations shift the λ_max_ in opposite directions by the same magnitude. These assumptions often fail because of the interactions among different amino acids, and conclusions derived from these mutagenesis experiments can be erroneous [27, 28, 29, 30]. To elucidate the correct mechanism of spectral tuning, it is imperative to genetically engineer and manipulate ancestral pigments by following the actual evolutionary processes in forward directions [27, 28, 29, 30]. This was done for RH1 [30], SWS1 [31], and M/LWS [32] pigments, but the similar molecular analyses have not been applied to the RH2 and SWS2 pigments.

Here, we inferred and genetically engineered the ancestral RH2 pigments at 13 critical stages of vertebrate evolution and determined their λ_max_s. Then, by introducing additional mutations, the molecular mechanisms of λ_max_ shifts at eight evolutionary steps of RH2 pigments have been established.

## Materials and methods

### Inference of ancestral sequences

**Fig. 1:**
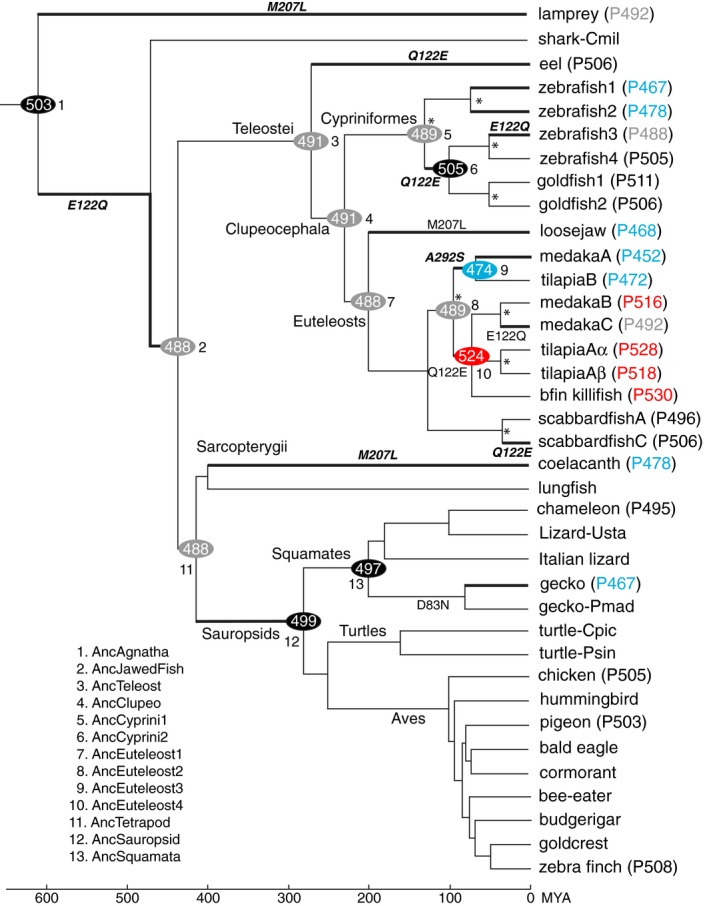
A composite tree topology of 37 representative RH2 pigments in vertebrates. The numbers in ovals and after P in parentheses show λ_max_s of the ancestral and present‐day pigments, respectively. Blue, grey, black and red indicate the λ_max_s of 452–478, 488–492, 495–511 and 516–530 nm, respectively. The critical amino acid substitutions are shown along branches. Asterisks (*) indicate the gene duplication events. Branches with significant λ_max_ shift (|λ_max_| ≥ 10 nm) are indicated by thick lines, and the amino acid changes that fully explain the λ_max_ shift are indicated by bold italics. The divergence times at the bottom are taken from the TimeTree of Life Web server (http://www.timetree.org).

The amino acid sequences of ancestral RH2 pigments were inferred by applying the phylogenetic analyses by maximum likelihood (PAML) with Jones–Taylor–Thornton (JTT), Whelan and Goldman (WAG), and Dayhoff amino acid substitution models [33] to the amino acid sequences of the 37 vertebrate RH2 pigments and those of the more restricted 24 pigments with known λ_max_s (Fig. 1), together with the RH1 pigment of bovine (*Bos taurus*) and the RH1, SWS1 and SWS2 pigments of lamprey (*Geotria australis*) as the outgroup (Table S1). Among the four paralogous groups of pigments, RH2 pigments are most closely related to RH1, SWS2 and SWS1 pigments, in that order [6, 34]. The divergence times of nonduplicated RH2 pigments were estimated from the TimeTree of Life Web server (http://www.timetree.org). The actual branch lengths of the composite phylogenetic tree were also determined by applying PAML to the user tree based on the amino acid sequences.

To infer the ancestral RH2 pigments, the amino acids between sites 31 and 311 were considered (Fig. S1A). In engineering ancestral pigments, we used an expression vector pMT5 that contained the amino (N) and carboxyl (C) termini (amino acids between positions 1–30 and 312–354, respectively) of the chameleon (P495; red letters in Fig. S1B) with the proper internal segment of a RH2 pigment. The N and C termini of chameleon (P495) do not affect the λ_max_ of a RH2 pigment significantly. For example, zebrafish4 (P505) with its own and the chameleon (P495) termini with the proper internal segment of a RH2 pigment have λ_max_s of 505 nm [16] and 507 nm, respectively, and gecko (P467) with its own and the chameleon termini have λ_max_s of 467 nm [35] and 466 nm, respectively. Similarly, different amino acids at the N and C termini do not modify the λ_max_s of SWS1 pigments [31].

Thirteen ancestral pigments, inferred from JTT model based on the 24 sequence data with more evenly distributed λ_max_s, were engineered: those of all vertebrates (AncAgnatha, node 1), jawed fishes (AncJawedFish, node 2), Teleostei (AncTeleost, node 3), Clupeocephala (AncClupeo, node 4), Cypriniformes (AncCyprini1–2, nodes 5 and 6), Euteleosts (AncEuteleost1–4, nodes 7–10), Tetrapods (AncTetrapod, node 11), Sauropsids (AncSauropsid, node 12) and Squamates (AncSquamata, node 13; Fig. 1). In this process, we engineered AncCyprini2 and AncSquamata first by introducing six and 12 mutations into zebrafish4 (P505) and chameleon (P495), respectively, and the remaining 11 ancestral pigments were engineered by sequentially introducing additional 128 mutations into various ancestral pigments.

All mutant opsins were generated by using QuikChange Site‐Directed Mutagenesis Kits (Stratagene, La Jolla, CA, USA). To rule out spurious mutations, the DNA fragments were sequenced by cycle sequencing reactions using the Sequitherm Excel II long‐read kits (Epicentre Technologies, Madison, WI, USA) with dye‐labelled M13 forward and reverse primers. Reactions were run on a LI‐COR (Lincoln, NE, USA) 4300LD automated DNA sequencer.

### The *in vitro* assay

Ancestral and other mutant opsins were expressed in COS1 cells by transient transfection [36]. The contiguous RH2 opsins between sites 31 and 311 were cloned into the *Eco*RI and *Sal*I restriction sites of the expression vector pMT5, which contained the N terminus (amino acids between positions 1 and 30) and C terminus (amino acids between positions 312 and 354) of the chameleon (P495; red letters in Fig. S1B). The visual pigments were regenerated by incubating the opsins with 11‐*cis*‐retinal (provided by R. K. Crouch at Storm Eye Institute, Medical University of South Carolina and the National Eye Institutes) and were purified using immobilized 1D4 (The Culture Center, Minneapolis, MN, USA) in buffer W1 (50 mm *N*‐(2‐hydroxyethyl) piperazine‐*N*′‐2‐ethanesulfonic acid (HEPES; pH 6.6), 140 mm NaCl, 3 mm MgCl_2_, 20% (w/v) glycerol and 0.1% dodecyl maltoside). UV‐visible spectra were recorded at 20 °C using a Hitachi U‐3000 dual beam spectrophotometer (LI‐COR Biosciences, Lincoln, NE, USA). Visual pigments were bleached for 3 min using a 60 W standard light bulb equipped with a Kodak Wratten #3 filter at a distance of 20 cm. Data were analysed using sigmaplot software (Jandel Scientific, San Rafael, CA, USA).

### Ethics

Research was carried out under approval of Emory University according to the university's animal ethics guidelines.

## Results

### The amino acid sequences of ancestral pigments

The ancestral amino acid sequences inferred are highly consistent. For example, when the earliest vertebrate ancestor AncAgnatha (node 1, Fig. 1) is considered, the amino acid sequences inferred by JTT and WAG models show that 241 (86%) out of a total of the 281 comparable sites are identical with Bayesian posterior probabilities (PPs) ≥ 0.95, 22 out of the remaining 40 sites also have identical amino acids with a 0.70 ≤ PP < 0.95, and different amino acids are predicted only at eight highly variable sites. The ancestral sequences inferred using the 24 and 37 sequences are also very similar. For AncAgnatha, JTT model shows that amino acids at 263 out of 281 sites (94%) are identical and only those at the other 18 sites differ (indicated by * in Table S2). At these 18 sites, the present‐day pigments have variable amino acids, but their λ_max_s are similar, which implies that these amino acids are not critical in determining the λ_max_s of visual pigments [34, 37].

At potentially critical sites 122, 207 and 292 [5], the 24 and 37 data sets predict identical amino acids throughout vertebrate evolution (Table S3). Specifically, E122Q, Q122E and M207L occurred three, four and two times, respectively, while A292S occurred once during the vertebrate evolution (Fig. 1).

One of these results disagrees with the published result. Previously, using PAML with JTT model, it was suggested that AncCyprini1 (node 5, Fig. 1; or Ancestor1 in Fig. S2A) had E122 with a PP of 0.77 [38], but our result using the identical method shows that AncCyprini1 (Fig. S2B) had Q122 with PPs of 0.89 and 0.97 using the 24 and 37 sequences, respectively (Table S3). Comparing the two data sets, we can find the cause of the discrepancy. That is, Chinen *et al*. used the teleost data consisting of 10 pigments with E122 and four pigments with Q122, which were heavily biased towards E122. On the other hand, our teleost data consist of eight pigments with E122 and nine pigments with Q122 (Fig. S2A,B, respectively). Because of the less‐biased data set and significantly higher PPs, it is most likely that AncCyprini1 had Q122.

### Functional differentiation

We applied the *in vitro* assay to the 13 ancestral pigments engineered. The results show that (a) the amino acid sequence of AncJawedFish differs from that of AncTetrapod at multiple sites (Fig. S1B), but these two pigments have identical λ_max_ values at 488 nm, and (b) the ratio of absorbance at ~ 280 nm to that at ~ 500 nm of the *in vitro* assay ranged from 2.5 (AncEuteleost) to 4.8 (AncSquamata; Fig. S3). In particular, AncAgnatha had a λ_max_ of 503 nm and its closest descendant, AncJawedFish (node 2, Fig. 1), decreased its λ_max_ to 488 nm, which had been maintained by six out of the remaining 11 ancestral pigments (nodes 3–5, 7, 8 and 11; grey ovals, Fig. 1; Fig. S3). However, the five others expanded their λ_max_s from 474 nm (node 9) to 524 nm (node 10) and the λ_max_s of present‐day pigments have been expanded further between ~ 450 and 530 nm.

We can find a total of 16 significant λ_max_ shifts (Δλ_max_ ≥ 10 nm), and three branches stand out with much larger λ_max_ shifts (|Δλ_max_| ≥ 30 nm): (a) AncEuteleost2 (P489) (node 8) → AncEuteleost4 (P524) (node 10); (b) AncEuteleost4 (P524) (node 10) → medakaC (P492); and (c) AncSquamata (P497) (node 13) → gecko (P467) (represented by thick branches; Fig. 1).

The λ_max_ shifts of RH2 pigments reveal three characteristics (Fig. 1). First, despite their similarities, the λ_max_s of zebrafish3 (P488) and medakaC (P492) did not evolve directly from that of AncJawedFish (P488), but these pigments reversed their λ_max_s to over 500 nm before reaching about 490 nm. These changes can be found only by reconstructing ancestral pigments at intermediate evolutionary steps. Second, with the exceptions of eel (P506), loosejaw (P468), coelacanth (P478) and gecko (P467), significant λ_max_ shifts have been generated by gene duplications followed by critical amino acid substitutions. Third, the decreased λ_max_s of loosejaw (P468) and coelacanth (P478) are closely connected with their unique adaptations to their highly species‐specific light environments ([39, 40, 41]) and that of gecko (P467) indicates the adaptation to its nocturnal environment.

Our mutagenesis results show that E122Q, Q122E, M207L and A292S shifted the λ_max_s of ancestral pigments by 7–20 nm (Table 1). These mutations explain eight evolutionary transitions: (a, b) AncAgnatha (P503) (node 1) → AncJawedFish (P488) (node 2) and AncCyprini2 (P505) (node 6) → zebrafish3 (P488) by E122Q; (c–e) AncTeleost (P491) (node 3) → eel (P506), AncCyprini1 (P489) (node 5) → AncCyprini2 (P505) (node 6), and AncEuteleost1 (P488) (node 7) → scabbardfishC (P506) by Q122E; (f, g) AncAgnatha (P503) (node 1) → lamprey (P492) and AncTetrapod (P488) (node 11) → coelacanth (P478) by M207L; and (h) AncEuteleost2 (P489) (node 8) → AncEuteleost3 (P478) (node 9) by A292S. However, the λ_max_ shifts of AncEuteleost1 (P488) (node 7) → Loosejaw (P468) and AncEuteleost4 (P524) (node 10)→ medakaC (P492) cannot be explained by M207L and E122Q alone, respectively. D83N can also shift the λ_max_s of other visual pigments by ~ 3–5 nm [5], but it does not shift the λ_max_ of AncSquamata (P497) (node 13; Table 1).

**Table 1:** Effects of various mutations on the λ_max_ shift. The numbers after P show λ_max_ values. When the λ_max_ values of the mutant and its descendant pigments are similar (|Δλ_max_| ≤ 4 nm), the results are shown by bold letters.

Ancestral pigment {node}	Mutation	λ_max_ (Δλ_max_) (nm)	Descendant {node}
**AncAgnatha (P503) {1}**	**E122Q**	**486 (−17)**	**AncJawedFish (P488) {2}**
**AncCyprini2 (P505) {6}**	**E122Q**	**489 (−16)**	**zebrafish3 (P488)**
**AncTeleost (P491) {3}**	**Q122E**	**508 (+17)**	**eel (P506)**
**AncCyprini1 (P489) {5}**	**Q122E**	**504 (+15)**	**AncCyprini2 (P505) {6}**
**AncEuteleost1 (P488) {7}**	**Q122E**	**503 (+15)**	**scabbardfishC (P506)**
**AncAgnatha (P503) {1}**	**M207L**	**493 (−10)**	**lamprey (P492)**
**AncTetrapod (P488) {11}**	**M207L**	**481 (−7)**	**coelacanth (P478)**
**AncEuteleost2 (P489) {8}**	**A292S**	**478 (−11)**	**AncEuteleost3 (P474) {9}**
AncEuteleost1 (P488) {7}	M207L	480 (−8)	Loosejaw (P468)
AncEuteleost4 (P524) {10}	E122Q	504 (−20)	medakaC (P492)
AncSquamata (P497) {13}	D83N	496 (−1)	gecko (P467)
AncEuteleost2 (P489) {8}	Q122E	504 (+15)	AncEuteleost4 (P524) {10}
AncEuteleost2 (P489) {8}	Y96T/Q122E/C213F	509 (+20)	AncEuteleost4 (P524) {10}
AncEuteleost2 (P489) {8}	V60F/F74Y/Y96T/Q122E/T209/C213F/I255V/L259M/A273G	510 (+21)	AncEuteleost4 (P524) {10}
AncTetrapod (P488) {11}	M44I	491 (+3)	AncSauropsid (P499) {12}
AncTetrapod (P488) {11}	L40V/A42C/M44I/I50T/V87A/A166S/I205L/L214I/L309M	491 (+3)	AncSauropsid (P499) {12}

We also searched for additional critical mutations along two branches: AncEuteleost2 (P489) (node 8) → AncEuteleost4 (P524) (node 10; Δλ_max_ = 35 nm) and AncTeptrapod (P488) (node 11) → AncSauropsid (P499) (node 12; Δλ_max_ = 11 nm; Fig. 1). For the first branch, the present‐day pigments descended from AncEuteleost2 (P489) can be distinguished into two groups: (a) medakaB (P516), tilapiaAα (P528), tilapiaAβ (P518) and bfin killifish (P530) with λ_max_ ≥ 516 nm; and (b) medakaA (P452), tilapiaB (P472) and medakaC (P492) with λ_max_ ≤ 492 nm (Fig. 1). Since nine changes (V60F, F74Y, Y96T, Q122E, T209V, C213F, I255V, L259M and A273G) are shared only by the pigments in the first group, we hypothesized that some of these mutations were responsible in increasing the λ_max_ of AncEuteleost4 (P524). But, Q122E, Y96T/Q122E/C213F and V60F/F74Y/Y96T/Q122E/T209V/C213F/I255V/L259M/A273G explain only 43% (15 nm), 57% (20 nm) and 60% (21 nm) of the observed λ_max_ shift (35 nm), respectively (Table 1). Hence, the interactions between E122Q and newly found critical changes Y96T and C213F do not improve the result significantly.

For the second branch, nine changes (L40V, A42C, M44I, I50T, V87A, A166S, I205L, L214I and L309M) are found in pigeon (P503), chicken (P505) and zebra finch (P508) with λ_max_ ≥ 503 nm, but are missing from coelacanth (P478) and gecko (P467) with λ_max_ ≤ 478 nm (Fig. 1). We found a new critical mutation (M44I), which increased the λ_max_ by 3 nm, but neither M44I nor all nine changes (L40V, A42C, M44I, I50T, V87A, A166S, I205L, L214I and L309M) explain the λ_max_ shift attained by AncSauropsid (P499) (Table 1).

These two examples reveal the complex nature of the spectral tunings in RH2 pigments. Chinen *et al*. [38] encountered the same problem in explaining the significant λ_max_ shift from Ancestor1 (P506) to Ancestor2 (P474; supplementary information, Fig. S2A; for more details, see Discussion section).

### Evolutionary rates of amino acid substitution

**Fig. 2:**
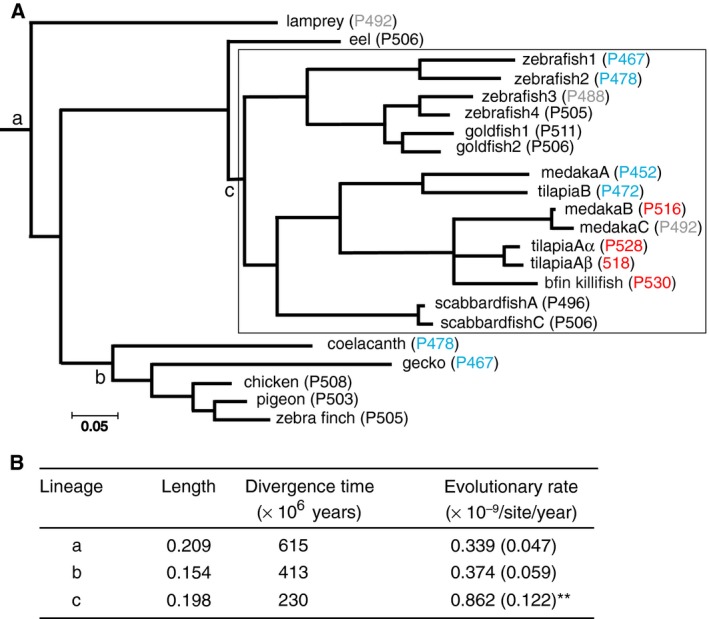
(A) The numbers of amino acid substitutions per site for a composite tree of 22 RH2 pigments were determined by applying PAML [33] to the amino acids between sites 31 and 311. Blue, grey, black and red indicate the λ_max_s of 452–478, 488–492, 495–511 nm and 516–530 nm, respectively. (B) The evolutionary rates of amino acid substitution along the branches including gene duplications (c lineage) and without gene duplications (a and b lineages). **P < 0.01 for the comparison of the branches between c‐present and a‐present and between those between c‐present and b‐present. The numbers in the parentheses indicate SE.

To evaluate the effects of gene duplication on the induction of the highly variable λ_max_s in teleost RH2 pigments, we considered representative 22 sequences and evaluated the numbers of amino acid substitutions per site per year at three lineages: (a) the vertebrate pigment lineage (lineage a), which excludes all Clupeocephala pigments (lineage c); (b) the Tetrapod pigment lineage (lineage b); and (c) the Clupeocephala pigment lineage where gene duplication events are prevalent (shown by a rectangle, lineage c; Fig. 2a). The branch lengths from these nodes to their descendant pigments were determined by applying PAML to the composite evolutionary tree of the amino acid sequences. The evolutionary rates were evaluated by taking the averages between bifurcated (or trifurcated) branches sequentially and assuming that lineages a, b and c originated 615, 230 and 413 million years ago (MYA), respectively (http://www.timetree.org). The results show that the evolutionary rate for lineage c (0.9 ± 0.12 × 10^−9^) is significantly higher than that for lineage a (0.3 ± 0.05 × 10^−9^; *Z* = 4.0) and lineage b (0.4 ± 0.06 × 10^−9^, *Z* = 3.6) at 1% level (Fig. 2b). Hence, the duplications of RH2 opsin genes were followed by the significantly accelerated amino acid substitutions, which led Clupeocephala RH2 pigments to expand the range of their λ_max_s.

## Discussion

Depending on whether AncCyprini1 (node 5, Fig. 1) had Q122 or E122, the evolutionary processes of zebrafish RH2 pigments can be interpreted very differently. If AncCyprini1 had E122 and a λ_max_ of 506 nm, the λ_max_ of zebrafish3 (P488) decreased by E122Q and Ancestor2 (P474) by E122Q and additional unidentified mutations [38] (Fig. S2A). On the other hand, if AncCyprini1 had Q122 and a λ_max_ of 489 nm and if the λ_max_ of Ancestor2 (P474) still holds, the λ_max_ shifts of AncCyprini2 (P505) and zebrafish3 (P488) are explained fully by Q122E and E122Q, respectively, but the critical mutations that caused the λ_max_ shift of Ancestor2 (P474) remain to be discovered (Fig. S2B).

This example shows that the mechanism of phenotypic adaptation can be understood by inferring the ancestral sequences correctly. For that, we need to use unbiased sequence data which, among other things, should consist of roughly equal numbers of molecules with different functions. Furthermore, to identify mutations that generated highly variable λ_max_s of ancestral and present‐day RH2 pigments, it would be necessary to construct various sets of chimeric pigments between a pair of pigments with different λ_max_s and then perform extensive mutagenesis experiments [42, 43, 44, 45].

The functional differentiation of euteleost RH2 pigments has been accelerated significantly by gene duplications. Evolution by gene duplication may be classified into four categories [46]: (a) gene duplication itself does not cause any selection, (b) the duplication itself renders selective advantage, (c) duplication occurs in a gene for which genetic variation already existed, and (d) duplications occur by whole‐genome duplication or large segmental duplication. The first category includes Ohno's neofunctionalization model [47] and various subfunctionalization models [48, 49, 50].

Among these possibilities, neither selection caused by gene duplication itself (category 2) nor functionally meaningful genetic variation (category 3) have been established in the preduplication phase of RH2 pigments. During fish evolution, Euteleost and Cypriniform ancestors appeared roughly 240 and 100 MYA, respectively [51]. Hence, RH2 gene duplications in fishes occurred much later than the fish‐specific whole‐genome duplication (WGD), which occurred about 350 MYA [52], showing that neither WGD nor large segmental duplication (category 4) [16] seem to be involved. The differential ontogenetic RH2 gene expression was described as ‘subfunctionalization’ [15], but actual dual functions of the ancestral RH2 gene have not been established. On the other hand, coelacanths (*Latimeria chalumnae*) seem to have moved into the depth at 200 m about 200 MYA [53] and the λ_max_s of the duplicated RH1 and RH2 pigments were decreased to 482 and 478 nm, respectively, and started to distinguish the narrow range of wavelengths available in its habitat [30, 54]. Therefore, the λ_max_ shifts of RH1 and RH2 pigments and newer duplicate RH2 pigments can be described best by the neofunctionalization model.

The accelerated evolutionary rates of the duplicate RH2 genes (lineage c, Fig. 2b) agree with the prediction [47, 55] and observations [56, 57, 58] of the evolution of duplicate genes, and not the decelerated evolutionary rates that were caused presumably by the more critical biological functions of duplicate genes, which cause purifying selection [59]. In general, the latter evolutionary patterns of duplicate genes do not apply to opsin genes because in order to move into different ecological environments, organisms need to readjust the λ_max_s of their paralogous visual pigments.

**Fig. 3:**
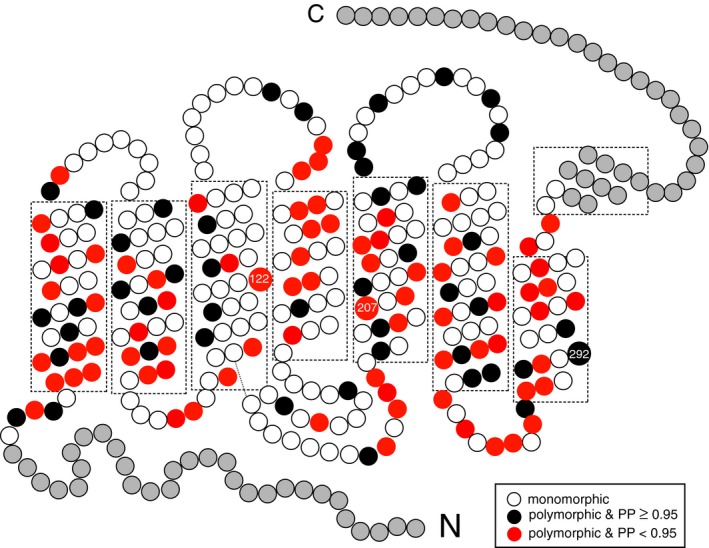
The neutral and adaptive mutations in ancestral pigments. Polymorphic sites with PP ≥ 0.95 and PP ≥ 0.70 are indicated by black and red circles, respectively, while white circles show monomorphic sites. Grey circles in the N and C termini indicate those of the anole RH2 pigment (see Materials and Methods). Amino acid changes at sites 122, 207 and 292 cause significant λ_max_ shifts. The 2‐D model is after Palczewski [60].

Finally, the λ_max_s of 10 out of the 13 ancestral pigments and lamprey (P492), eel (P506), zebrafish3 (P488), scabbardfishC (P506) and coelacanth (P478) can be explained fully by the amino acid changes E122Q, Q122E, M207L and A292S. When the amino acids at the comparable 281 sites (positions 31–311) of these 15 pigments are compared, 123 sites are polymorphic (red and black circles, respectively, Fig. 3). Hence, for these pigments, the amino acid changes only at sites 122, 207 and 292 (2.4%) out of the 123 sites cause λ_max_ shifts and the remaining changes can be regarded as ‘selectively neutral’. Slightly higher magnitudes (~ 5%) of adaptive sites have been observed for RH1 [30], SWS1 [31] and M/LWS [32] pigments.

## Conclusions

By inferring and engineering the RH2 pigments at 13 critical stages of vertebrate evolution, we have shown that the green‐sensitive pigments of the vertebrate ancestor had a λ_max_ of 503 nm, from which the 12 ancestral pigments changed their λ_max_s between 474 and 524 nm and the present‐day RH2 pigments have further expanded the range to ~ 450–530 nm. Eight out of the 16 significant λ_max_ shifts of RH2 pigments can be explained by the mutations E122Q (twice), Q122E (thrice), M207L (twice) and A292S (once). The highly variable λ_max_s of teleost RH2 pigments have been achieved by gene duplications followed by accelerated amino acid substitution.

## Conflict of interest

The authors declare no conflict of interest.

## Author contributions

SY designed research, performed phylogenetic analyses and wrote the paper. HJ performed experiments and commented on the manuscript.

## Supporting information


**Fig. S1.** Aligned amino acid sequences of RH2 pigments. (**A**) Present‐day pigments, where Bkillifish, scabbard, and Ital lizard are bluefin killifish, scabbardfish, and Italian lizard, respectively. The numbers after P in parentheses show λ_max_s. Amino acids sites 122, 207, and 292 are indicated by stars (*). (**B**) 13 ancestral pigments inferred by applying the PAML with JTT model to the 24 sequence data, where the ancestral amino acids with < PP of 95% or less are indicated by bold italic letters. The amino acids in red letters are those of chameleon (P495). Following the tradition in vision science, the amino acid site numbers are those of bovine RH1 (GenBank accession no. M21606).
**Fig. S2.** Two different inferences of the RH2 pigment evolution in Cypriniformes. The AncCyprini1 was inferred to have either E122 (**A,** Chinen et al. [38]) or Q122 (**B**, present analysis). The λ_max_s of Ancestors 1–3 are taken from (Chinen et al. [38]). The numbers in ovals and after P in rectangles show λ_max_s of the ancestral and present‐day pigments, respectively. The amino acids at site 122 are given at the right column. E122Q decreases the λ_max_, whereas Q122E increases λ_max_. E122Q* explains about 47% of the λ_max_ shift of Ancestor2. Blue, grey, black, and red indicates the λ_max_s of 452–478, 488–492, 495–511, and 516–530 nm, respectively.
**Fig. S3.** Absorption spectra of ancestral RH2 pigments. The λ_max_ values of AncJawedFish and AncTetrapod are identical at 488 nm but their absorbance levels at ~ 280 nm are 1.1 and 1.6, respectively.
**Table S1.** The source of RH2 pigment sequences.
**Table S2.** Amino acids of AncAgnatha with PP < 0.95 (in parentheses) inferred using PAML with JTT model.
**Table S3.** Amino acids of ancestral pigments at three critical sites with PP (in parentheses) inferred using PAML with JTT substitution model.

## References

[1] Wald G (1935) Carotenoids and the visual cycle. J Gen Physiol 19, 351–371. 19872932 10.1085/jgp.19.2.351 PMC2141433

[2] Wald G (1955) The photoreceptor process in vision. Am J Ophthalmol 40, 18–41. 13268550 10.1016/0002-9394(55)91835-3

[3] Walls GL (1942) The Vertebrate Eye and its Adaptive Radiation. Cranbrook Institute of Science, Bloomfield Hills, MI.

[4] Lythgoe JN (1979) The Ecology of Vision. Clarendon, Oxford.

[5] Yokoyama S (2008) Evolution of dim‐light and color visual pigments. Annu Rev Genom Human Genet 9, 259–282. 10.1146/annurev.genom.9.081307.164228 18544031

[6] Yokoyama S (2000) Molecular evolution of vertebrate visual pigments. Prog Retin Eye Res 19, 385–419. 10785616 10.1016/s1350-9462(00)00002-1

[7] Ebrey T and Koutalos Y (2001) Vertebrate photoreceptors. Prog Retin Eye Res 20, 49–94. 11070368 10.1016/s1350-9462(00)00014-8

[8] Munz FW and McFarland WN (1977) Evolutionary adaptations of fishes to the photic environment In Handbook of Sensory Physiology (Crescitelli F, ed), pp. 193–274. Springer‐Verlag, Berlin.

[9] McFarland WN and Munz FW (1975) Part II: the photic environment of clear tropical seas during the day. Vision Res 15, 1063–1070. 1166605 10.1016/0042-6989(75)90002-4

[10] McFarland WN and Munz FW (1975) Part III: the evolution of photopic visual pigments in fishes. Vision Res 15, 1071–1080. 1166606 10.1016/0042-6989(75)90003-6

[11] Jerlov NG (1976) Marine Optics. Elsevier, Amsterdam.

[12] Musilova Z , Cortesi F , Matschiner M , Davies WIL , Suresh Patel JS , Stieb SM , de Busserolles F , Malmstrøm M , Tørresen OK , Brown CJ *et al* (2019) Vision using multiple distinct rod opsins in deep‐sea fishes. Science 364, 588–592. 31073066 10.1126/science.aav4632 PMC6628886

[13] Johnson RL , Grant KB , Zankel TC , Boehm MF , Merbs SL , Nathans J and Nakanishi K (1993) Cloning and expression of goldfish opsin sequences. Biochemistry 32, 208–14. 8418840 10.1021/bi00052a027

[14] Matsumoto Y , Fukamachi S , Mitani H and Kawamura S (2006) Functional characterization of visual opsin repertoire in Medaka (Oryzias latipes). Gene 371, 268–278. 16460888 10.1016/j.gene.2005.12.005

[15] Spady TC , Parry JW , Robinson PR , Hunt DM , Bowmaker JK and Carleton KL (2006) Evolution of the cichlid visual palette through ontogenetic subfunctionalization of the opsin gene arrays. Mol Biol Evol 23, 1538–1547. 16720697 10.1093/molbev/msl014

[16] Chinen A , Hamaoka T , Yamada Y and Kawamura S (2003) Gene duplication and spectral diversification of cone visual pigments of zebrafish. Genetics 163, 663–675. 12618404 10.1093/genetics/163.2.663 PMC1462461

[17] Yokoyama S and Tada T (2010) Evolutionary dynamics of rhodopsin type 2 opsins in vertebrates. Mol Biol Evol 27, 133–141. 19759234 10.1093/molbev/msp217 PMC2794311

[18] Yokoyama S , Takenaka N and Blow N (2007) A novel spectral tuning in the short wavelength‐sensitive (SWS1 and SWS2) pigments of bluefin killifish (Lucania goodei). Gene 396, 196–202. 17498892 10.1016/j.gene.2007.03.019 PMC1963460

[19] Korner KE , Schlupp I , Plath M and Loew ER (2006) Spectral sensitivity of mollies: comparing surface‐ and cave‐dwelling Atlantic mollies, *Poecilia mexicana* . J Fish Biol 69, 54–65.

[20] Archer SN and Lythgoe JN (1990) The visual pigment basis for cone polymorphism in the guppy, *Poecilia reticulate* . Vision Res 30, 225–33. 2309457 10.1016/0042-6989(90)90038-m

[21] Watson CT , Gray SM , Hoffmann M , Lubieniecki KP , Joy JB , Sandkam BA , Weigel D , Loew E , Dreyer C , Davidson WS *et al* (2011) Gene duplication and divergence of long wavelength‐sensitive opsin genes in the guppy, *Poecilia reticulate* . J Mol Evol 72, 240–252. 21170644 10.1007/s00239-010-9426-z

[22] Kawamura S , Kasagi S , Kasai D , Tezuka A , Shoji A , Takahashi A , Imai H and Kawata M (2016) Spectral sensitivity of guppy visual pigments reconstituted *in vitro* to resolve association of opsins with cone cell types. Vision Res 127, 67–73. 27476645 10.1016/j.visres.2016.06.013

[23] Jordan R , Kellogg K , Howe D , Juanes F , Stauffer J and Loew E (2006) Photopigment spectral absorbance of Lake Malawi cichlids. J Fish Biol 68, 1291–1299.

[24] Carleton KL , Parry JW , Bowmaker JK , Hunt DM and Seehausen O (2005) Colour vision and speciation in Lake Victoria cichlids of the genus *Pundamilia* . Mol Ecol 14, 4341–4353. 16313597 10.1111/j.1365-294X.2005.02735.x

[25] Parry JW , Carleton KL , Spady T , Carboo A , Hunt DM and Bowmaker JK (2005) Mix and match color vision: tuning spectral sensitivity by differential opsin gene expression in Lake Malawi cichlids. Curr Biol 15, 1734–1739. 16213819 10.1016/j.cub.2005.08.010

[26] Kasagi S , Mizusawa K , Murakami N , Andoh T , Furufuji S , Kawamura S and Takahashi A (2015) Molecular and functional characterization of opsins in barfin flounder (Verasper moseri). Gene 556, 182–191. 25433330 10.1016/j.gene.2014.11.054

[27] Yokoyama S , Tada T , Liu Y , Faggionato D and Altun A (2016) A simple method for studying the molecular mechanisms of ultraviolet and violet reception in vertebrates. BMC Evol Biol 16, 64. 27001075 10.1186/s12862-016-0637-9 PMC4802639

[28] Yokoyama S (2012) Synthesis of experimental molecular biology and evolutionary biology: an example from the world of vision. Bioscience 62, 939–948. 23483186 10.1525/bio.2012.62.11.3 PMC3593118

[29] Yokoyama S (2013) Synthetic biology of phenotypic adaptation in vertebrates: the next frontier. Mol Biol Evol 30, 1495–1499. 23603936 10.1093/molbev/mst075 PMC3684858

[30] Yokoyama S , Tada T , Zhang H and Britt L (2008) Elucidation of phenotypic adaptations: molecular analyses of dim‐light vision proteins in vertebrates. Proc Nat Acad Sci USA 105, 13480–13485. 18768804 10.1073/pnas.0802426105 PMC2533215

[31] Shi Y and Yokoyama S (2003) Molecular analysis of the evolutionary significance of ultraviolet vision in vertebrates. Proc Natl Acad Sci USA 100, 8308–8313. 12824471 10.1073/pnas.1532535100 PMC166225

[32] Yokoyama S and Radlwimmer FB (2001) The molecular genetics and evolution of red and green color vision in vertebrates. Genetics 158, 1697–1710. 11545071 10.1093/genetics/158.4.1697 PMC1461741

[33] Yang Z (2007) PAML 4: phylogenetic analysis by maximum likelihood. Mol Biol Evol 24, 1586–1591. 17483113 10.1093/molbev/msm088

[34] Yokoyama S and Yokoyama R (1996) Adaptive evolution of photoreceptors and visual pigments in vertebrates. Annu Rev Ecol Syst 27, 543–567.

[35] Takenaka N and Yokoyama S (2007) Mechanisms of spectral tuning in the RH2 pigments of Tokay gecko and American chameleon. Gene 399, 26–32. 17590287 10.1016/j.gene.2007.04.036 PMC2693072

[36] Yokoyama S (2000) Phylogenetic analysis and experimental approaches to study color vision in vertebrates In Methods Enzymol(Palczewski K, ed), pp. 312–25. Academic Press, San Diego, CA. 10.1016/s0076-6879(00)15851-3 10736710

[37] Yokoyama S (1997) Molecular genetic basis of adaptive selection: examples from color vision in vertebrates. Annu Rev Genet 31, 315–336. 9442898 10.1146/annurev.genet.31.1.315

[38] Chinen A , Matsumoto Y and Kawamura S (2005) Reconstitution of ancestral green visual pigments of zebrafish and molecular mechanism of their spectral differentiation. Mol Biol Evol 22, 1001–1010. 15647516 10.1093/molbev/msi086

[39] Schliewen U , Fricke H , Shartl M , Epplen JT and Paabo S (1993) Which home for coelacanth? Nature 363, 405.

[40] Fricke H , Hissmann K , Schauer J and Plante R (1995) Yet more danger for coelacanths. Nature 374, 314.7885468

[41] Herring PJ and Cope C (2005) Red bioluminescence in fishes: on the suborbital photophores of Malacosteus, Pachystomias and Aristostomias. Mar Biol 148, 383–394.

[42] Takahashi Y and Yokoyama S (2005) Genetic basis of spectral tuning in the violet‐sensitive visual pigment of African clawed frog, *Xenopus laevis* . Genetics 171, 1153–1160. 16079229 10.1534/genetics.105.045849 PMC1456818

[43] Yokoyama S , Altun A , Jia H , Yang H , Koyama T , Faggionato D , Liu Y and Starmer WT (2015) Adaptive evolutionary paths from UV reception to sensing violet light by epistatic interactions. Sci Adv 1, e1500162. 26601250 10.1126/sciadv.1500162 PMC4643761

[44] Shi Y , Radlwimmer FB and Yokoyama S (2001) Molecular genetics and the evolution of ultraviolet vision in vertebrates. Proc Natl Acad Sci USA 98, 11731–11736. 11573008 10.1073/pnas.201257398 PMC58798

[45] Sun H , Macke JP and Nathans J (1997) Mechanisms of spectral tuning in the mouse green cone pigment. Proc Natl Acad Sci USA 94, 8860–8865. 9238068 10.1073/pnas.94.16.8860 PMC23167

[46] Innan H and Kondrashov F (2010) The evolution of gene duplications: classifying and distinguishing between models. Nature Rev Genet 11, 97–108. 20051986 10.1038/nrg2689

[47] Ohno S (1970) Evolution by Gene Duplication. Springer‐Verlag, New York, NY.

[48] Lynch M and Force A (2000) The probability of duplicate gene preservation by subfunctionalization. Genetics 154, 459–473. 10629003 10.1093/genetics/154.1.459 PMC1460895

[49] Hughes AL (1994) The evolution of functionally novel proteins after gene duplication. Proc R Soc Lond B 256, 119–124. 10.1098/rspb.1994.0058 8029240

[50] Näsvall J , Sun L , Roth JR and Andersson DI (2012) Real‐time evolution of new genes by innovation, amplification, and divergence. Science 338, 384–387. 23087246 10.1126/science.1226521 PMC4392837

[51] Near TJ , Eytan RI , Dornburg A , Kuhn KL , Moore JA , Davis MP , Wainwright PC , Friedman M and Smith WL (2012) Resolution of ray‐finned fish phylogeny and timing of diversification. Proc Natl Acad Sci USA 109, 13698–13703. 22869754 10.1073/pnas.1206625109 PMC3427055

[52] Meyer A and Peer Y (2005) From 2R to 3R: evidence for a fish‐specific genome duplication (FDGD). BioEssays 27, 937–945. 16108068 10.1002/bies.20293

[53] Yokoyama S and Tada T (2000) Adaptive evolution of the African and Indonesian coelacanths to deep‐sea environments. Gene 261, 35–42. 11164035 10.1016/s0378-1119(00)00474-1

[54] Yokoyama S , Zhang H , Radlwimmer FB and Blow NS (1999) Adaptive evolution of color vision of the Comoran coelacanth (*Latimeria chalumnae*). Proc Natl Acad Sci USA 96, 6279–6284. 10339578 10.1073/pnas.96.11.6279 PMC26872

[55] Ohno S (1973) Ancient linkage groups and frozen accidents. Nature 244, 259–262. 4200792 10.1038/244259a0

[56] Kondrashov FA , Rogozin IB , Wolf YI and Koonin EV (2002) Selection in the evolution of gene duplications. Genome Biol 3, RESEARCH0008. 11864370 10.1186/gb-2002-3-2-research0008 PMC65685

[57] Lynch M and Conery JS (2000) The evolutionary fate and consequences of duplicate genes. Science 290, 1151–1155. 11073452 10.1126/science.290.5494.1151

[58] Li C , Li M , Dunwell JM and Zhang Y‐M (2012) Gene duplication and an accelerated evolutionary rate in 11S globulin genes are associated with higher protein synthesis in dicots as compared to monocots. MBC Evol Biol 12, 15. 10.1186/1471-2148-12-15 PMC3305549 22292855

[59] Jordan IK , Wolf YI and Koonin EV (2004) Duplicated genes evolve slower than singletons despite the initial rate increase. MBC Evol Biol 4, 22. 10.1186/1471-2148-4-22 PMC481058 15238160

[60] Palczewski K (2006) G protein‐coupled receptor rhodopsin. Annu Rev Biochem 75, 743–767. 16756510 10.1146/annurev.biochem.75.103004.142743 PMC1560097

