# Supplementary material for: Origin and adaptation of green‐sensitive (RH2) pigments in vertebrates
Source: FEBS Open Bio. 2020 Apr 11;10(5):873–82. doi: 10.1002/2211-5463.12843 (PMC7193153; doi:10.1002/2211-5463.12843)
Supplement: Supplementary file 1 — Fig. S1. Aligned amino acid sequences of RH2 pigments. (A) Present‐day pigments, where Bkillifish, scabbard, and Ital lizard are bluefin killifish, scabbardfish, and Italian lizard, respectively. The numbers after P in parentheses show λmaxs. Amino acids sites 122, 207, and 292 are indicated by stars (*). (B) 13 ancestral pigments inferred by applying the PAML with JTT model to the 24 sequence data, where the ancestral amino acids with < PP of 95% or less are indicated by bold italic letters. The amino acids in red letters are those of chameleon (P495). Following the tradition in vision science, the amino acid site numbers are those of bovine RH1 (GenBank accession no. M21606). Fig. S2. Two different inferences of the RH2 pigment evolution in Cypriniformes. The AncCyprini1 was inferred to have either E122 (A, Chinen et al. [38]) or Q122 (B, present analysis). The λmaxs of Ancestors 1–3 are taken from (Chinen et al. [38]). The numbers in ovals and after P in rectangles show λmaxs of the ancestral and present‐day pigments, respectively. The amino acids at site 122 are given at the right column. E122Q decreases the λmax, whereas Q122E increases λmax. E122Q* explains about 47% of the λmax shift of Ancestor2. Blue, grey, black, and red indicates the λmaxs of 452–478, 488–492, 495–511, and 516–530 nm, respectively. Fig. S3. Absorption spectra of ancestral RH2 pigments. The λmax values of AncJawedFish and AncTetrapod are identical at 488 nm but their absorbance levels at ~ 280 nm are 1.1 and 1.6, respectively. Table S1. The source of RH2 pigment sequences. Table S2. Amino acids of AncAgnatha with PP < 0.95 (in parentheses) inferred using PAML with JTT model. Table S3. Amino acids of ancestral pigments at three critical sites with PP (in parentheses) inferred using PAML with JTT substitution model. [file FEB4-10-873-s001.pdf]

Supporting information for

Origin and adaptive evolution of green-sensitive (RH2) pigments in vertebrates

Shozo Yokoyama and Huiyong Jia

Shozo Yokoyama
Email: syokoya@emory.edu

Contents:

Tables S1 to S3
Figs. S1 to S3

Table S1. The source of RH2 pigment sequences.

Pigment	Species	GenBank
lamprey (P492)†	*Geotria australis*	AY366494
shark-Cmil	*Callorhinchus milii*	EF565168
eel (P506)†	*Anguilla anguilla*	FJ515778
zebrafish 1 (P467)†	*Danio rerio*	AB087805
zebrafish 2 (P478)†	*Danio rerio*	AB087806
zebrafish 3 (P488)†	*Danio rerio*	AB087807
zebrafish 4 (P505)†	*Danio rerio*	AB087808
goldfish 1 (P511)†	*Carassius auratus auratus*	L11865
goldfish 2 (P506)†	*Carassius auratus auratus*	L11866
loosejaw (P468)†	*Aristostomias scintillans*	KT933073
medaka A (P452)†	*Oryzias latipes*	AB223053
medaka B (P516)†	*Oryzias latipes*	AB223054
medaka C (P492)†	*Oryzias latipes*	AB223055
tilapiaAα (P528)†	*Oreochromis niloticus*	DQ235683
tilapiaAβ (P518)†	*Oreochromis niloticus*	DQ235682
tilapiaB (P472)†	*Oreochromis niloticus*	DQ235681
bfin killifish (P530)†	*Lucania goodei*	AY296739
scabbardfish A (P496)†	*Lepidopus fitchi*	GQ414752
scabbardfish C (P506)†	*Lepidopus fitchi*	GQ421594
coelacanth (P478)†	*Latimeria chalumnae*	AH007713
lungfish	*Neoceratodus forsteri*	EF526296
chameleon (P495)†	*Anolis carolinensis*	AF134189
lizard-Usta	*Uta stansburiana*	DQ100324
Italian lizard	*Podarcis sicula*	AY941829
gecko (P467)†	*Gekko gecko*	M92035
turtle-Cpic	*Chrysemys picta bellii*	XM_005309675
turtle-Psin	*Pelodiscus sinensis*	XM_006119345
chicken (P505)†	*Gallus gallus*	M92038
hummingbird	*Calypte anna*	XM_008499430
pigeon (P503)†	*Columba livia*	AF149232
bald eagle	*Haliaeetus leucocephalus*	XM_010584680
cormorant	*Phalacrocorax carbo*	XM_009505908
bee-eater	*Merops nubicus*	XM_008939766
budgerigar	*Melopsittacus undulatus*	XM_005142831
goldcrest	*Regulus regulus*	KM977595
zebra finch (P508)†	*Taeniopygia guttata*	AF222330
lamprey RH1	*Geotria australis*	U67123
bovine RH1	*Bos taurus*	M21606
lamprey SWS1	*Geotria australis*	AY366495
lamprey SWS2	*Geotria australis*	AY366492

† 24 RH2 pigments with known λ_{max}s.

The numbers after P in parentheses are λ_{max}s.

Table S2. Amino acids of AncAgnatha with PP <0.95 (in parentheses) inferred using PAML with JTT model.

Site	24 pigments		37 pigments		Site	24 pigments		37 pigments	
36*	K (0.93)	Q (0.04)	K (0.67)	M (0.17)	173*	F (0.92)	V (0.07)	V (0.97)	F (0.03)
37	Y (0.57)	F (0.43)	Y (0.90)	F (0.10)	197	K (0.87)	N (0.09)	K (0.65)	N (0.23)
39*	A (0.74)	V (0.24)	V (0.74)	I (0.09)	199*	H (0.79)	N (0.16)	N (0.88)	H (0.11)
41*	A (0.83)	S (0.16)	S (0.67)	A (0.33)	205	I (0.55)	M (0.42)	I (0.90)	M (0.10)
50*	I (0.46)	V (0.40)	V (0.55)	I (0.23)	207	M (0.91)	L (0.09)	M (0.80)	L (0.20)
54	I (0.63)	V (0.37)	I (0.64)	V (0.35)	214*	L (0.83)	I (0.11)	I (0.98)	L (0.02)
83	D (0.90)	N (0.10)	D (0.85)	N (0.15)	222*	T (0.89)	S (0.11)	S (0.96)	T (0.04)
85	F (0.93)	I (0.05)	F (0.80)	I (0.12)	256	I (0.62)	L (0.37)	I (0.61)	L (0.38)
88*	C (0.41)	L (0.32)	M (0.88)	L (0.10)	259	I (0.53)	V (0.42)	I (0.94)	V (0.06)
93*	V (0.78)	T (0.21)	T (0.60)	L (0.26)	271	V (0.85)	F (0.09)	V (0.77)	F (0.20)
98	A (0.85)	S (0.15)	A (0.83)	S (0.16)	279*	K (0.94)	R (0.06)	K (0.81)	Q (0.18)
111	N (0.45)	S (0.29)	N (0.98)	S (0.01)	281*	A (0.50)	S (0.43)	S (0.59)	A (0.36)
122	E (0.78)	Q (0.22)	E (0.87)	Q (0.13)	282	D (0.94)	E (0.04)	D (0.75)	E (0.18)
124*	A (0.96)	S (0.04)	S (0.79)	A (0.21)	284*	S (0.52)	T (0.34)	S (0.97)	G (0.03)
139	V (0.94)	I (0.06)	V (0.76)	I (0.24)	287	L (0.53)	F (0.30)	L (0.59)	F (0.40)
149*	A (0.65)	S (0.21)	G (0.94)	S (0.04)	299*	A (0.95)	S (0.03)	A (0.94)	V (0.06)
150	S (0.92)	N (0.04)	S (0.94)	N (0.06)	304	I (0.92)	V (0.08)	I (1.0)	-
154	I (0.96)	L (0.02)	I (0.88)	L (0.10)	307	V (0.92)	I (0.08)	V (0.51)	I (0.49)
157	V (0.90)	I (0.10)	V (0.95)	I (0.05)	309*	L (0.58)	M (0.42)	M (1.0)	-
162*	I (0.62)	V (0.38)	V (0.94)	I (0.06)					

*The amino acids inferred based on 24 and 37 data sets differ.

Table S3. Amino acids of ancestral pigments at three critical sites with PP (in parentheses) inferred using PAML with JTT substitution model.

		Amino acid sites					
Pigment	Data set	122		207		292	
1. AncAgnatha	24	E (0.78)	Q (0.22)	M (0.91)	L (0.09)	A (1.0)	-
	37	E (0.87)	Q (0.13)	M (0.80)	L (0.20)	A (1.0)	-
2. AncJawedfish	24	Q (0.86)	E (0.14)	M (0.97)	L (0.03)	A (1.0)	-
	37	Q (0.95)	E (0.05)	M (0.82)	L (0.18)	A (1.0)	-
3. AncTeleost	24	Q (0.84)	E (0.16)	M (1.0)	-	A (1.0)	-
	37	Q (0.96)	E (0.04)	M (1.0)	-	A (1.0)	-
4. AncClupeo	24	Q (0.89)	E (0.11)	M (1.0)	-	A (1.0)	-
	37	Q (0.97)	E (0.03)	M (1.0)	-	A (1.0)	-
5. AncCyprini1	24	Q (0.89)	E (0.11)	M (1.0)	-	A (1.0)	-
	37	Q (0.97)	E (0.03)	M (1.0)	-	A (1.0)	-
6. AncCyprini2	24	E (0.75)	Q (0.25)	M (1.0)	-	A (1.0)	-
	37	E (0.76)	Q (0.24)	M (1.0)	-	A (1.0)	-
7. AncEuteleost1	24	Q (0.97)	E (0.03)	M (1.0)	-	A (1.0)	-
	37	Q (1.0)	-	M (1.0)	-	A (1.0)	-
8. AncEuteleost2	24	Q (0.97)	E (0.03)	M (1.0)	-	A (0.96)	S (0.04)
	37	Q (1.0)	-	M (1.0)	-	A (0.96)	S (0.04)
9. AncEuteleost3	24	Q (1.0)	-	M (1.0)	-	S (0.98)	A (0.02)
	37	Q (1.0)	-	M (1.0)	-	S (0.97)	A (0.03)
10. AncEuteleost4	24	E (1.0)	-	M (1.0)	-	A (1.0)	-
	37	E (1.0)	-	M (1.0)	-	A (1.0)	-
11. AncTetrapod	24	Q (0.99)	E (0.01)	M (0.97)	L (0.03)	A (1.0)	-
	37	Q (0.98)	E (0.02)	M (1.0)	-	A (1.0)	-
12. AncSauropsid	24	Q (1.0)	-	M (1.0)	-	A (1.0)	-
	37	Q (1.0)	-	M (1.0)	-	A (1.0)	-
13. AncReptile	24	Q (1.0)	-	M (1.0)	-	A (1.0)	-
	37	Q (1.0)	-	M (1.0)	-	A (1.0)	-

Fig. S1

(A)

```
                          40        50        60        70        80        90
lamprey(P492)     LADPWMYSAISAYVFTLILIGFPVNFMTLFVTFKLKKLRQPLNFILVNLCVADLLMIMFG
shark-Cmil        LADRWLFSSISAYMFLLICAGLPINGLTLLVTVKHKKLRQPLNYILVNLAVANLVMIMFG
eel(P506)         LGEPWQYKMLAVYVFFLICFGFPINGLTLVVTAQHKKLRQPLNFILVNLAVAGLIMCIFG
goldfish1(P511)   LAEPWQFKILALYLFFLMSMGLPINGLTLVVTAQHKKLRQPLNFILVNLAVAGTIMVCFG
goldfish2(P506)   LAEPWQFKLLAVYMFFLICLGLPINGLTLICTAQHKKLRQPLNFILVNLAVAGAIMVCFG
zebrafish1(P467)  LAEPWKFKALAFYMFLLIIFGFPINVLTLVVTAQHKKLRQPLNYILVNLAFAGTIMVIFG
zebrafish2(P478)  LADPWQFKALAFYMFFLICFGLPINVLTLLVTAQHKKLRQPLNYILVNLAFAGTIMAFFG
zebrafish3(P488)  LAEPWQFKLLAVYMFFLMCFGFPINGLTLVVTAQHKKLRQPLNFILVNLAVAGTIMVCFG
zebrafish4(P505)  LAEPWQFKLLAVYMFFLICLGFPINGLTLLVTAQHKKLRQPLNFILVNLAVAGTIMVCFG
medakaA(P452)     LADPWQFKLLGIYMFFLILTGFPINALTLVVTAQNKKLRQPLNFILVNLAVAGLIMVCFG
medakaB(P516)     MVDPIMYKILAFYMFFLICTGTPINGLTLYVTATNKKLQQPLNFILVNLAVAGLIMCAFG
medakaC(P492)     MVDPIMYKILAFYMFFLICTGTPINGLTLYVTATNKKLQQPLNFILVNLAVAGLIMCAFG
Bkillifish(P530)  MVDPMIYKVLAFYMFFLICTGTPINGLTLFVTAQNKKLRQPLNYILVNLAVAGLIMCCFG
tilapiaAa(P528)   MVDPITYKILAFYMFFLICTGTPINGLTLFVTAQNKKLRQPLNYILVNLAVAGLIMCCFG
tilapiaAb(P518)   LADPIFFKLLAFYMFFLICTGTPINGLTLFVTAQNKKLRQPLNYILVNLAVAGLIMCCFG
tilapiaB(P472)    LADPWFFKLLAFYMVFLVITGFPINFLTLLVTAQNKKLRQPLNFILVNLAVAGLIMVMFG
scabbardA(P496)   LGAPWQFKLLALYMFFLISFGFPINALTLVVTAQHKKLRQPLNFILVNLAVAGLVMVCFG
scabbardC(P506)   FGAPWQFKLLALYMFFLISFGFPINALTLVVTAQHKKLRQPLNFILVNLAVAGLVMVCFG
loosejaw          LGPPWQFYALAAYMFMLICFGLPINVLTLLVTAQNKKLRQPLNFILVNLAVAGTIMVVFG
lungfish          LADPWKYSIVCAYMFFLIITGLPINLLTLVVTFKHKKLRQPLNYILVNLAVADLFMVCFG
coelacanth(P478)  LAEPWKFSVLCAYMFLLIILGFPINFLTLLVTFKHKKLRQPLNYILVNLAVASLFMVVFG
chameleon(P495)   LAEPWKYKVVCCYIFFLIFTGLPINILTLLVTFKHKKLRQPLNYILVNLAVADLFMACFG
gecko(P467)       LADPWKFKVLSFYMFFLIAAGMPLNGLTLFVTFQHKKLRQPLNYILVNLAAANLVTVCCG
geckoPmad         LAEPWKFKALSLYMFFLILVGLPLNGLTLFVTFQHKKLRQPLNYILVNLAVANLLMVICG
lizardUsta        LAEPWKYKIVCCYIFFLISTGLPINILTLLVTFKHKKLRQPLNYILVNLAVADLFMACFG
pigeon(P503)      LAEPWKYRVVCCYIFFLISTGLPINLLTLLVTFKHKKLRQPLNYILVNLAVADLFMACFG
zebrafinch(P508)  LAEPWKYRLVCCYIFFLISTGFPINFLTLLVTFKHKKLRQPLNYILVNLAVADLCMACFG
chicken(P505)     LAEPWKYRLVCCYIFFLISTGLPINLLTLLVTFKHKKLRQPLNYILVNLAVADLFMACFG
budgeriger        LAEPWKYRVVCCYIFFLISTGLPINLLTLLVTFKHKKLRQPLNYILVNLAVADLFMACFG
Ital lizard       LAEPWKYKMVCCYIFFLISTGLPINLLTLLVTFKHKKLRQPLNYILVNLAVADLFMACFG
bald eagle        LAEPWKYRVVCCYIFFLISTGLPINLLTLLVTFKHKKLRQPLNYILVNLAVADLFMACFG
bee-eater         LAEPWKYRLVCCYIFFLISTGLPINLLTLLVTFKHKKLRQPLNYILVNLAVADLFMACFG
cormorant         LAEPWKYRLVCCYIFFLISTGLPINLLTLLVTFKHKKLRQPLNYILVNLAVADLLMACFG
turtleCpic        LAEPWKYRIVCCYIFFLIFTGLPINLLTLLVTFKHKKLRQPLNYILVNLAVADLFMACFG
turtlePsin        LAEPWKYRIVCCYIFFLIFTGLPINLLTLLVTFKHKKLRQPLNYILVNLAVADLFMACFG
goldcrest         LAEPWKYRLVCCYIFFLISTGFPINFLTLLVTFKHKKLRQPLNYILVNLAVADLCMACFG
hummingbird       LAEPWKYRLVCCYIFFLISTGLPINLLTLLVTFKHKKLRQPLNYILVNLAVADLFMACFG
```

Fig. S1. Aligned amino acid sequences of RH2 pigments. (**A**) Present-day pigments, where Bkillifish, scabbard, and Ital lizard are bluefin killifish, scabbardfish, and Italian lizard, respectively. The numbers after P in parentheses show λ_{max}s. Amino acids sites 122, 207, and 292 are indicated by stars (*). (**B**) 13 ancestral pigments inferred by applying the PAML with JTT model to the 24 sequence data, where the ancestral amino acids with < PP of 95% or less are indicated by bold italic letters. The amino acids in red letters are those of chameleon (P495). Following the tradition in vision science, the amino acid site numbers are those of bovine rhodopsin (GenBank accession no. M21606).

(Fig. S1A, continued)

```
                          100       110       120 *     130       140       150
lamprey(P492)      FTTTFYTAMNGYFVFGPTGCNIEGFFATLGGEVSLWSLVMLAIERYIVVCKPMGNFRFAT
shark-Cmil         FVLSFYTTMNGYFIFGPIGCIFEGFFATLGGQVALWSLVVLAIERYIVICKPMGNFRFGT
eel(P506)          FTITFYTAINGYFVFGPVGCAVEGFMATLGGEVALWSLVVLAIERYIVVCKPMGSFKFTS
goldfish1(P511)    FTVTFYTAINGYFVLGPTGCAVEGFMATLGGEVALWSLVVLAIERYIVVCKPMGSFKFSS
goldfish2(P506)    FTVTFYTAINGYFALGPTGCAVEGFMATLGGEVALWSLVVLAIERYIVVCKPMGSFKFSS
zebrafish1(P467)   FTVSFYCSLVGYMALGPLGCVMEGFFATLGGQVALWSLVVLAIERYIVVCKPMGSFKFSA
zebrafish2(P478)   FTVTFYCSINGYMALGPTGCAIEGFFATLGGQVALWSLVVLAIERYIVVCKPMGSFKFSS
zebrafish3(P488)   FTVTFYTAINGYFVLGPTGCAIEGFMATLGGQISLWSLVVLAIERYIVVCKPMGSFKFSS
zebrafish4(P505)   FTVTFYTAINGYFVLGPTGCAIEGFMATLGGEVALWSLVVLAVERYIVVCKPMGSFKFSA
medakaA(P452)      FTVCIYSCMVGYFSLGPLGCTIEGFMATLGGQVSLWSLVVLAIERYIVVCKPMGSFKFTA
medakaB(P516)      FTITITSSFYGYFVLGPTFCAIEGFMATLGGEVALWSLVVLAVERYIVVCKPMGSFKFSG
medakaC(P492)      FTITLNSSFYGYFVLGPTFRAVEGFMATLGGQIALWSLVVLAVERYIVVCKPMGSFKFTG
Bkillifish(P530)   FTITITSAVNGYFILGPTFCGIEGFMATLGGEVALWSLVVLAIERYIVVCKPMGSFKFTG
tilapiaAa(P528)    FTITITSAVNGYFILGPTFCAIEGFMATLGGEVALWSLVVLAVERYIVVCKPMGSFKFTG
tilapiaAb(P518)    FTITITSAINGYFVLGTTFCAIEGFMATLGGEVALWSLVVLAVERYIVVCKPMGSFKFTG
tilapiaB(P472)     FTVTIYSCLNGYFSLGPLSCAIEGFMATVGGQVSLWSLVVLAVERYIVVCKPMGSFKFTA
scabbardA(P496)    FTGTIIITALNGYFIFGPLGCAIEGFMATLGGQVALWSLVVLAVERYIVVCKPMGSFKFGT
scabbardC(P506)    FTGTIIITALNGYFIFGPLGCAIEGFVATLGGQVALWSLVVLAVERYIVVCKPMGSFKFGT
loosejaw           FTITFISSLNGYFVFGTLGCTVEGFMATLGGQVSLWSLVVLAIERYIVVCKPMGSFKFGA
lungfish           FTVTFSTAINGYFIFGPRGCAIEGFMATLGGEVALWSLVVLAIERYIVVCKPMGNFRFSN
coelacanth(P478)   FTVTFYSSLNGYFVLGPMGCAMEGFFATLGGQVALWSLVVLAIERYIVVCKPMGNFRFAS
chameleon(P495)    FTVTFYTAWNGYFIFGPIGCAIEGFFATLGGQVALWSLVVLAIERYIVVCKPMGNFRFSA
gecko(P467)        FTVTFYASWYAYFVFGPIGCAIEGFFATIGGQVALWSLVVLAIERYIVICKPMGNFRFSA
geckoPmad          FTVTFYTSWYGYFVFGPMGCAFEGFFATIGGQVALWSLVVLAIERYIVICKPMGNFRFSS
lizardUsta         FTVTFYTAWNGYFIFGPIGCAIEGFFATLGGQVALWSLVVLAIERYIVVCKPMGNFRFSA
pigeon(P503)       FTVTFYTAWNGYFVFGPVGCAIEGFFATLGGQVALWSLVVLAIERYIVVCKPMGNFRFSA
zebrafinch(P508)   FTVTFYTAWNGYFVFGPIGCAVEGFFATLGGQVALWSLVVLAIERYIVICKPMGNFRFSA
chicken(P505)      FTVTFYTAWNGYFVFGPVGCAVEGFFATLGGQVALWSLVVLAIERYIVVCKPMGNFRFSA
budgeriger         FTVTFYTAWNGYFVFGPIGCAVEGFFATLGGQVALWSLVVLAIERYIVVCKPMGNFRFSS
Ital lizard        FTVTFYTAWNGYFIFGPIGCAIEGFFATLGGQVALWSLVVLAIERYIVVCKPMGNFRFSS
bald eagle         FTVTFYTAWNGYFVFGPVGCAVEGFFATLGGQVALWSLVVLAIERYIVVCKPMGNFRFSA
bee-eater          FTVTFYTAWNGYFVFGPAGCAVEGFFATLGGQVALWSLVVLAIERYIVVCKPMGNFRFSA
cormorant          FTVTFYTAWNGYFVFGPVGCAVEGFFATLGGQVALWSLVVLAIERYIVVCKPMGNFRFSA
turtleCpic         FTVTFYTAWNGYFIFGPIGCAVEGFFATLGGQVALWSLVVLAIERYIVVCKPMGNFRFSA
turtlePsin         FTVTFYTAWNGYFIFGPTGCAVEGFFATLGGQVALWSLVVLAIERYIVVCKPMGNFRFSA
goldcrest          FTVTFYTAWNGYFVFGPIGCAVEGFFATLGGQVALWSLVVLAIERYIVICKPMGNFRFSA
hummingbird        FTVTFYTAWNGYFVFGPIGCAVEGFFATLGGQVALWSLVVLAIERYIVVCKPMGNFRFSA
```

(Fig. S1A, continued)

```
                         160       170       180       190       200      *210
lamprey(P492)     THAALGVVFTWVMASACAVPPLVGWSRYIPEGMQCSCGPDYYTLNPKYYNESYVIYLFLV
shark-Cmil        SHALMGMGFTWFMALTAAVPPLVGWSRFIPEGFQCSCTPDFYTTNPLYNNDSYLMYLFSV
eel(P506)         THAIVGIAFTWIMALSCAAPPLFGWSRYMPEGLQCSCGPDYYTLNPDYHNESYVIYMFVV
goldfish1(P511)   SHAFAGIAFTWVMALACAAPPLFGWSRYIPEGMQCSCGPDYYTLNPDYNNESYVIYMFVC
goldfish2(P506)   THASAGIAFTWVMAMACAAPPLVGWSRYIPEGIQCSCGPDYYTLNPEYNNESYVLYMFIC
zebrafish1(P467) NHAMAGIAFTWFMACSCAVPPLFGWSRYLPEGMQTSCGPDYYTLNPEYNNESYVMYMFSC
zebrafish2(P478) NHAMAGIAFTWVMASSCAVPPLFGWSRYIPEGMQTSCGPDYYTLNPEFNNESYVLYMFSC
zebrafish3(P488) NHAFAGIGFTWIMALSCAAPPLVGWSRYIPEGMQCSCGPDYYTLNPDYNNESYVLYMFCC
zebrafish4(P505) SHAFAGCAFTWVMAMACAAPPLVGWSRYIPEGMQCSCGPDYYTLNPEYNNESYVLYMFIC
medakaA(P452)     THSAAGCAFTWIMASSCAVPPLVGWSRYIPEGIQVSCGPDYYTLAPGFNNESFVMYMFTC
medakaB(P516)     THAGAGVLLTWVMALACAAPPLCGWSRYIPEGMQCSCGPDYYTLAPGFNNESYVIYMFVV
medakaC(P492)     THAAAGVLFTWVMALACAAPPLLGWSRYIPEGMQCSCGPDYYTLAPGFNNESYVMYMFCV
Bkillifish(P530) THAAAGVLSTWIMALACAAPPLFGWSRYIPEGMQCSCGPDYYTLAPGFNNESYVIYMFVV
tilapiaAa(P528)   AHAGAGVFFTWVMAMACAAPPLFGWSRYIPEGMQCSCGPDYYTLAPGFNNESYVIYMFVV
tilapiaAb(P518)   AHAGAGVLFTWIMAMACAAPPLFGWSRYIPEGMQCSCGPDYYTLAPGFNNESYVIYMFVV
tilapiaB(P472)    THAGVGCAFTWIMAMSCAAPPLLGWSRYIPEGIQVSCGPDYYTLAPGYNNESYVMYMFTC
scabbardA(P496)   SHAAAGVIFTWIMAMACAAPPLFGWSRYLPEGLQCSCGPDYYTNSPGFNNDSYIIYMFTC
scabbardC(P506)   SHAAAGVIFTWIMAMACAAPPLFGWSRYLPEGLQCSCGPDYYTNSPGFNNDSYIIYMFTC
loosejaw          SHAAAGVMFTWIMAFSCAAPPLFGWSRFIPEGLQVSCGPDYYTLNPTYNNESYVMYLFTC
lungfish          NHSIIGIVFTWLAALSCAAPPLFGWSRYLPEGMQCSCGPDYYTMNPDYHNESFVIYMFVV
coelacanth(P478) SHAIMGIAFTWIMALACAAPPLVGWSRYIPEGLQCSCGPDYYTLNPDFHNESYVMYLFLV
chameleon(P495)   THALMGISFTWFMSFSCAAPPLLGWSRYIPEGMQCSCGPDYYTLNPDYHNESYVLYMFGV
gecko(P467)       THAIMGIAFTWFMALACAGPPLFGWSRFIPEGMQCSCGPDYYTLNPDFHNESYVIYMFIV
geckoPmad         SHAMMGISFTWFMALCCGGPPLFGWSRFIPEGMQCSCGPDYYTLNPDFHNESYVIYLFTV
lizardUsta        THALLGIAFTWFMSFSCAAPPLFGWSRYIPEGMQCSCGPDYYTLNPDYHNESYVLYMFLI
pigeon(P503)      SHAMMGIAFTWIMAFSCAAPPLFGWSRYMPEGMQCSCGPDYYTHNPDYHNESYVLYMFII
zebrafinch(P508) SHALMGIAFTWVMAISCAAPPLFGWSRYIPEGMQCSCGPDYYTHNPDFHNESYVLYMFVI
chicken(P505)     THAMMGIAFTWVMAFSCAAPPLFGWSRYMPEGMQCSCGPDYYTHNPDYHNESYVLYMFVI
budgeriger        SHAMMGIAFTWVMAFSCAAPPLFGWSRYMPEGMQCSCGPDYYTHNPDYHNESYVLYMFVI
Ital lizard       SHALMGIAFTWVMSLSCACPPLFGWSRYIPEGMQCSCGPDYYTLNPDYHNESYVVYMFVI
bald eagle        THAMMGIAFTWIMAFSCAAPPLFGWSRYMPEGMQCSCGPDYYTHNPDYHNESYVLYMFVI
bee-eater         SHAMMGIAFTWVMAFSCAAPPLFGWSRYMPEGMQCSCGPDYYTHNPDYHNESYVLYMFLI
cormorant         THAMMGIAFTWVMAFSCATPPLFGWSRYMPEGMQCSCGPDYYTHNPDYHNESYVLYMFII
turtleCpic        THALMGISFTWAMAFSCAAPPLFGWSRYIPEGMQCSCGPDYYTLNPDYHNESYVVYMFMV
turtlePsin        THALMGISFTWVMAFSCAAPPLFGWSRYIPEGMQCSCGPDYYTLNPDYHNESYVVYMFMG
goldcrest         SHAMMGIAFTWVMAISCAAPPLFGWSRYIPEGMQCSCGPDYYTHNPDFHNESYVLYMLVI
hummingbird       SHAMMGIAFTWVMAFSCAAPPLFGWSRYMPEGMQCSCGPDYYTHNPDYHNESYVLYMFVI
```

(Fig. S1A, continued)

```
                              220       230       240       250       260       270
lamprey(P492)     HFLLPVTIIFFTYGRLICTVKEAAAQQQESASTQKAEREVTRMVIIMVVGFLVCWVPYAS
shark-Cmil        HFAFPVTLIFFSYGRLICKVKEAAAQQQESATTQKAEKEVTRMVILMVIGFLTAWLPYAS
eel(P506)         HFFLPVLIIFFAYGSLVCTVKAAAATQQESASTQKAEKEVTRMVILMVLGFMVAWTPYAT
goldfish1(P511)   HFILPVAVIFFTYGRLVCTVKAAAAQQQDSASTQKAEREVTKMVILMVFGFLIAWTPYAT
goldfish2(P506)   HFILPVTIIFFTYGRLVCTVKAAAAQQQDSASTQKAEREVTKMVILMVLGFLVAWTPYAT
zebrafish1(P467)  HFCIPVTTIFFTYGSLVCTVKAAAAQQQESESTQKAEREVTRMVILMVLGFLFAWVPYAS
zebrafish2(P478)  HFCVPVTTIFFTYGSLVCTVKAAAAQQQESESTQKAEREVTRMVILMVLGFLVAWVPYAS
zebrafish3(P488)  HFIFPVTTIFFTYGRLVCTVKAAAAQQQESESTQKAEREVTRMVILMVLGFLVAWTPYAS
zebrafish4(P505)  HFILPVTIIFFTYGRLVCTVKAAAAQQQESESTQKAEREVTRMVILMVLGFLIAWTPYAT
medakaA(P452)     HFCVPVFTIFFTYGSLVMTVKAAAAQQQDSASTQKAEKEVTRMCFLMVLGFLLAWVPYAS
medakaB(P516)     HFFVPVFLIFFTYGSLVLTVKAAAAQQQDSASTQKAEKEVTRMCLLMVFGFLVAWVPYAS
medakaC(P492)     HFCIPVFLIFFTYGSLVLTVKAAAAQQQDSASTQKAEKEVTRMCLLMVFGFLVAWVPYAS
Bkillifish(P530)  HFFIPVFLIFFTYGSLVMTVKAAAAQQQDSASTQKAEKEVTRMCVLMVMGFLIAWTPYAT
tilapiaAa(P528)   HFFVPVFIIFFTYGSLVMTVKAAAAQQQDSASTQKAEKEVTRMCVLMVMGFLVAWTPYAS
tilapiaAb(P518)   HFFVPVFIIFFTYGSLVMTVKAAAAQQQDSASTQKAEKEVTRMCVLMVMGFLIAWTPYAS
tilapiaB(P472)    HFCVPVFTIFFTYGNLVFTVKAAASQQQDSASTQKAEKEVTRMCILMVLGFLFAWTPYAS
scabbardA(P496)   HFCFPVSVIFFTYGSLVLTVKAAAASQQESASTQKAEREVTRMCVLMVFGFLLAWTPYAS
scabbardC(P506)   HFCFPVFVIFFTYGSLVLTVKAAAASQKESASTQKAEREVTRMCVLMVFGFLLAWTPYAS
loosejaw          HFCIPVTTIFFTYGSLVCTVKAAAASQQESESTQKAEREVTRMCVLMVMGFLVAWVPYAS
lungfish          HFFIPVIVIFVSYGRLICKVKEAAAQQQESASTQKAEREVTRMVILMVIGFMTAWTPYAT
coelacanth(P478)  HFLLPITTIFFTYGRLICKVKEAAAQQQESASTQKAEKEVTRMVILMVIGFLTAWVPYAS
chameleon(P495)   HFVIPVVVIFFSYGRLICKVREAAAQQQESASTQKAEREVTRMVILMVLGFLLAWTPYAM
gecko(P467)       HFTVPMVVIFFSYGRLVCKVREAAAQQQESATTQKAEKEVTRMVILMVLGFLLAWTPYAA
geckoPmad         HFLTPMIIIFFSYGRLVCKVREAAAQQQESATTQKAEKEVTRMVILMVMGFLVAWTPYAT
lizardUsta        HFVIPVVIIFFSYGRLICKVREAAAQQQESASTQKAEKEVTRMVILMVLGFLLAWTPYAV
pigeon(P503)      HFIIPVVVIFFSYGRLICKVREAAAQQQESATTQKAEKEVTRMVILMVLGFMLAWTPYAV
zebrafinch(P508)  HFIIPVVIIFFSYGRLVCKVREAAAQQQESATTQKAEKEVTRMVILMVLGFMLAWTPYAV
chicken(P505)     HFIIPVVVIFFSYGRLICKVREAAAQQQESATTQKAEKEVTRMVILMVLGFMLAWTPYAV
budgeriger        HFIIPVVVIFFSYGRLICKVREAAAQQQESATTQKAEREVTRMVILMVLGFMLAWTPYAV
Ital lizard       HFVIPVVVIFFSYGRLICKVREAAAQQQESASTQKAEKEVTRMVILMVLGFMLAWTPYAV
bald eagle        HFIIPVVVIFFSYGRLICKVREAAAQQQESATTQKAEKEVTRMVILMVLGFMLAWTPYAV
bee-eater         HFIIPVVVIFFSYGRLICKVREAAAQQQESATTQKAEKEVTRMVILMVLGFMLAWTPYAV
cormorant         HFIIPVTVIFFSYGRLICKVREAAAQQQESATTQKAEKEVTRMVILMVLGFMLAWTPYAV
turtleCpic        HFVVPVVVIFFSYGRLICKVREAAAQQQESASTQKAEREVTRMVILMVLGFLMAWTPYAL
turtlePsin        HFVIPVVIIFFSYGQLICKVREAAAQQQESASTQKAEREVTRMVILMVLGFLLAWTPYAV
goldcrest         HFIIPVVIIFFSYGRLVCKVREAAAQQQESATTQKAEKEVTRMVILMVLGFMLAWTPYAV
hummingbird       HFIIPVVVIFFSYGRLICKVREHALSFLESATTQKAEKEVTRMVILMVLGFMLAWTPYAV
```

(Fig. S1A, continued)

```
                                280       290 *     300       310
Lamprey(P492)     FAFYLFMNKGILFSATAMTVPAFFSKSSVLYNPIIYVLLNK
shark-Cmil        LSIWIFTHQGAWISPLLMTIPSFFSKSSVLYNPIIYILMNK
eel(P506)         MTGYIFLNKGVAFTPQSMAVPAFFSKSSALYNPVIYVLLNK
goldfish1(P511)   VAAWIFFNKGADFSAKFMAIPAFFSKSSALYNPVIYVLLNK
goldfish2(P506)   VAAWIFFNKGAAFSAQFMAIPAFFSKTSALYNPVIYVLLNK
zebrafish1(P467)  FAAWIFFNRGAAFSAQAMAVPAFFSKTSAVFNPIIYVLLNK
zebrafish2(P478)  FAAWIFFNRGAAFSAQAMAIPAFFSKASALFNPIIYVLLNK
zebrafish3(P488)  VAAWIFFNRGAAFSAQFMAVPAFFSKSSSIFNPIIYVLLNK
zebrafish4(P505)  VAAWIFFNKGAAFSAQFMAVPAFFSKTSALYNPVIYVLLNK
medakaA(P452)     YAAWIFFNRGAAFSAMSMAIPSFFSKSSALFNPIIYILLNK
medakaB(P516)     FAGWIFLNKGASFTALTASIPAFFAKSSALYNAVIYVLLNK
medakaC(P492)     FAAWIFLNKGASFTALTASIPAFFAKSSALYNAVIYVLLNK
Bkillifish(P530)  FAGWIFLNKGAAFTALTAALPAFFAKSSALYNPVIYVLMNK
tilapiaAa(P528)   FAGWIFLNKGAAFSALTAALPAFFAKSSALYNPVIYVLMNK
tilapiaAb(P518)   FAGWIFLNKGAAFSALTAAIPAFFAKSSALYNPVIYVLMNK
tilapiaB(P472)    FAAWIFFNKGAAFTATAMAIPSFFSKSSALFNPIIYILMNK
scabbardA(P496)   LTAYIFMNKGVAFTPQSMAVPAFFAKSSALFNPIIYVLLNK
scabbardC(P506)   LTAYIFMNKGVAFTPQSMAVPAFFAKSSALFNPIIYVLLNK
loosejaw          FAAYIFFNKGVAFTAQSMAVPAFFSKSSALFNPIIYVLMNK
lungfish          VAFWIFMNKGAEFGATFMAAPAFFSKSSALYNPIIYVLMNK
coelacanth(P478)  AAFWIFCNRGAEFTATLMTVPAFFSKSSCLFNPIIYVLLNK
chameleon(P495)   VAFWIFTNKGVDFSATLMSVPAFFSKSSSLYNPIIYVLMNK
gecko(P467)       TAIWIFTNRGAAFSVTFMTIPAFFSKSSSIYNPIIYVLLNK
geckoPmad         VACWIFNNKGAEFSVTFMTVPAFFSKSSCIYNPIIYVLLNK
lizardUsta        VAFWIFTNKGADFSATLMSVPAFFSKSSSLYNPIIYVLMNK
pigeon(P503)      VAFWIFTNKGADFTATLMAVPAFFSKSSSLYNPIIYVLMNK
zebrafinch(P508)  VAFWIFTNKGADFTATLMAVPAFFSKSSSLYNPIIYVLMNK
chicken(P505)     VAFWIFTNKGADFTATLMAVPAFFSKSSSLYNPIIYVLMNK
budgeriger        VAFWIFTNKGADFSATLMSVPAFFSKSSSLYNPVIYVLMNK
Ital lizard       VAFWIFTNKGADFSATLMSVPAFFSKSSSLYNPIIYVLMNK
bald eagle        VAFWIFTNKGADFTATLMSVPAFFSKSSSLYNPIIYVLMNK
bee-eater         VAFWIFTNKGADFSATLMSVPAFFSKSSSLYNPIIYVLMNK
cormorant         VAFWIFTNKGADFTATLMAVPAFFSKSSSLYNPIIYFLSLL
turtleCpic        VAFWIFTNKGADFSATLMSVPAFFSKSSSLYNPVIYVLMNK
turtlePsin        VAFWIFTNKGADFSATLMSVPAFFSKSSSLYNPIIYVLMNK
goldcrest         VAFWIFTNKGADFSATLMAVPAFFSKSSSLYNPIIYVLMNK
hummingbird       VAFWIFTNKGADFTATLMSVPAFFSKSSSLYNPIIYVLMNK
```

(Fig. S1B)

(B)

```
                            10        20        30        40        50        60
AncAgnatha(24) MNGTEGINFYVPLSNKTGLVRSPFEYPQYYLAEPWKYSALAAYMFFLILIGFPINFLTLF
          (37) MNGTEGINFYVPLSNKTGLVRSPFEYPQYYLAEPWKYSVLSAYMFFLILVGFPINFLTLF
AncJawedFi(24) MNGTEGINFYVPLSNKTGLVRSPFEYPQYYLAEPWKYSVLAAYMFFLILIGFPINFLTLL
          (37) MNGTEGINFYVPLSNKTGLVRSPFEYPQYYLAEPWKYSVLSAYMFFLICTGFPINFLTLL
AncTeleost(24) MNGTEGINFYVPLSNKTGLVRSPFEYPQYYLAEPWQYKLLAVYMFFLICFGFPINGLTLV
          (37) MNGTEGINFYVPLSNKTGLVRSPFEYPQYYLAEPWQYKLLAVYMFFLICFGFPINGLTLV
AncClupeoc(24) MNGTEGINFYVPLSNKTGLVRSPFEYPQYYLAEPWQFKLLAVYMFFLICFGFPINGLTLV
          (37) MNGTEGINFYVPLSNKTGLVRSPFEYPQYYLAEPWQFKLLAVYMFFLICFGFPINGLTLV
AncCyprin1(24) MNGTEGINFYVPLSNKTGLVRSPFEYPQYYLAEPWQFKLLAVYMFFLICFGFPINGLTLV
          (37) MNGTEGINFYVPLSNKTGLVRSPFEYPQYYLAEPWQFKLLAVYMFFLICFGFPINGLTLV
AncCyprin2(24) MNGTEGINFYVPLSNKTGLVRSPFEYPQYYLAEPWQFKLLAVYMFFLICLGFPINGLTLV
          (37) MNGTEGINFYVPLSNKTGLVRSPFEYPQYYLAEPWQFKLLAVYMFFLICLGFPINGLTLV
AncEutelo1(24) MNGTEGINFYVPLSNKTGLVRSPFEYPQYYLAEPWQFKLLAVYMFFLICFGFPINGLTLV
          (37) MNGTEGINFYVPLSNKTGLVRSPFEYPQYYLAEPWQFKLLAVYMFFLICFGFPINALTLV
AncEutelo2(24) MNGTEGINFYVPLSNKTGLVRSPFEYPQYYLADPWQFKLLAFYMFFLICTGFPINALTLV
          (37) MNGTEGINFYVPLSNKTGLVRSPFEYPQYYLADPWQFKLLAFYMFFLICTGFPINALTLV
AncEutelo3(24) MNGTEGINFYVPLSNKTGLVRSPFEYPQYYLADPWQFKLLAFYMFFLILTGFPINALTLV
          (37) MNGTEGINFYVPLSNKTGLVRSPFEYPQYYLADPWQFKLLAFYMFFLILTGFPINALTLV
AncEutelo4(24) MNGTEGINFYVPLSNKTGLVRSPFEYPQYYMVDPIIYKILAFYMFFLICTGTPINGLTLF
          (37) MNGTEGINFYVPLSNKTGLVRSPFEYPQYYMVDPIIYKILAFYMFFLICTGTPINGLTLF
AncTetrapo(24) MNGTEGINFYVPLSNKTGLVRSPFEYPQYYLAEPWKYSVLCAYMFFLILIGFPINFLTLL
          (37) MNGTEGINFYVPLSNKTGLVRSPFEYPQYYLAEPWKYSIVCAYMFFLICTGFPINFLTLL
AncSaurops(24) MNGTEGINFYVPLSNKTGLVRSPFEYPQYYLAEPWKYKVVCCYIFFLISTGLPINFLTLL
          (37) MNGTEGINFYVPLSNKTGLVRSPFEYPQYYLAEPWKYKIVCCYIFFLISTGLPINLLTLL
AncSquamat(24) MNGTEGINFYVPLSNKTGLVRSPFEYPQYYLAEPWKYKVVCCYIFFLISTGLPINFLTLL
          (37) MNGTEGINFYVPLSNKTGLVRSPFEYPQYYLAEPWKYKIVCCYIFFLISTGLPINLLTLL

                            70        80        90       100       110       120
AncAgnatha(24) VTFKHKKLRQPLNYILVNLAVADLFMVCFGFTVTFYTAMNGYFVFGPTGCNIEGFFATLG
          (37) VTVKHKKLRQPLNYILVNLAVADLFMVMFGFTTTFYTAMNGYFVFGPTGCNIEGFFATLG
AncJawedFi(24) VTFKHKKLRQPLNYILVNLAVADLFMVCFGFTVTFYTAMNGYFVFGPTGCAIEGFFATLG
          (37) VTVKHKKLRQPLNYILVNLAVADLFMVMFGFTVTFYTAMNGYFVFGPIGCAIEGFFATLG
AncTeleost(24) VTAQHKKLRQPLNFILVNLAVAGLIMVCFGFTVTFYTAINGYFVFGPTGCAIEGFMATLG
          (37) VTAQHKKLRQPLNFILVNLAVAGLIMVCFGFTVTFYTAINGYFVFGPIGCAIEGFMATLG
AncClupeoc(24) VTAQHKKLRQPLNFILVNLAVAGLIMVCFGFTVTFYTAINGYFVFGPTGCAIEGFMATLG
          (37) VTAQHKKLRQPLNFILVNLAVAGLIMVCFGFTVTFYTAINGYFVFGPIGCAIEGFMATLG
AncCyprin1(24) VTAQHKKLRQPLNFILVNLAVAGTIMVCFGFTVTFYTAINGYFVLGPTGCAIEGFMATLG
          (37) VTAQHKKLRQPLNFILVNLAVAGTIMVCFGFTVTFYTAINGYFVLGPTGCAIEGFMATLG
AncCyprin2(24) VTAQHKKLRQPLNFILVNLAVAGTIMVCFGFTVTFYTAINGYFVLGPTGCAIEGFMATLG
          (37) VTAQHKKLRQPLNFILVNLAVAGTIMVCFGFTVTFYTAINGYFVLGPTGCAIEGFMATLG
AncEutelo1(24) VTAQHKKLRQPLNFILVNLAVAGLIMVCFGFTVTFYTALNGYFVFGPLGCAIEGFMATLG
          (37) VTAQHKKLRQPLNFILVNLAVAGLIMVCFGFTVTFYTALNGYFVFGPLGCAIEGFMATLG
AncEutelo2(24) VTAQNKKLRQPLNFILVNLAVAGLIMVCFGFTVTIYSALNGYFVLGPLGCAIEGFMATLG
          (37) VTAQNKKLRQPLNFILVNLAVAGLIMVCFGFTVTIYSALNGYFILGPLGCAIEGFMATLG
AncEutelo3(24) VTAQNKKLRQPLNFILVNLAVAGLIMVCFGFTVTIYSCLNGYFSLGPLGCAIEGFMATLG
          (37) VTAQNKKLRQPLNFILVNLAVAGLIMVCFGFTVTIYSCLNGYFSLGPLGCAIEGFMATLG
AncEutelo4(24) VTAQNKKLRQPLNYILVNLAVAGLIMCCFGFTITITSAVNGYFVLGPTFCAIEGFMATLG
          (37) VTAQNKKLRQPLNYILVNLAVAGLIMCCFGFTITITSAVNGYFILGPTFCAIEGFMATLG
AncTetrapo(24) VTFKHKKLRQPLNYILVNLAVADLFMVCFGFTVTFYTAMNGYFVFGPTGCAIEGFFATLG
          (37) VTFKHKKLRQPLNYILVNLAVADLFMVCFGFTVTFYTAINGYFIFGPIGCAIEGFFATLG
AncSaurops(24) VTFKHKKLRQPLNYILVNLAVADLFMACFGFTVTFYTAWNGYFVFGPIGCAIEGFFATLG
          (37) VTFKHKKLRQPLNYILVNLAVADLFMACFGFTVTFYTAWNGYFIFGPIGCAIEGFFATLG
AncSquamat(24) VTFKHKKLRQPLNYILVNLAVADLFMACFGFTVTFYTAWNGYFVFGPIGCAIEGFFATLG
          (37) VTFKHKKLRQPLNYILVNLAVADLFMACFGFTVTFYTAWNGYFIFGPIGCAIEGFFATLG
```

(Fig. S1B, continued)

```
                    130       140       150       160       170       180
AncAgnatha(24) GEVALWSLVVLAIERYIVVCKPMGNFRFASTHAIMGVAFTWIMALACAAPPLFGWSRYIP
          (37) GEVSLWSLVVLAIERYIVVCKPMGNFRFGSTHAIMGVAFTWVMALSCAAPPLVGWSRYIP
AncJawedFi(24) GQVALWSLVVLAIERYIVVCKPMGNFRFASTHAIMGIAFTWIMALACAAPPLFGWSRYIP
          (37) GQVALWSLVVLAIERYIVVCKPMGNFRFGSTHAIMGIAFTWVMALSCAAPPLVGWSRYIP
AncTeleost(24) GQVALWSLVVLAIERYIVVCKPMGSFKFTSTHAIVGIAFTWIMALSCAAPPLFGWSRYIP
          (37) GQVALWSLVVLAIERYIVVCKPMGSFKFSSTHAIVGIAFTWIMALSCAAPPLFGWSRYIP
AncClupeoc(24) GQVALWSLVVLAIERYIVVCKPMGSFKFSSTHAIAGIAFTWIMAMSCAAPPLFGWSRYIP
          (37) GQVALWSLVVLAIERYIVVCKPMGSFKFSSSHAIAGIAFTWIMAMSCAAPPLFGWSRYIP
AncCyprin1(24) GQVALWSLVVLAIERYIVVCKPMGSFKFSSSHAIAGIAFTWVMAMSCAAPPLFGWSRYIP
          (37) GQVALWSLVVLAIERYIVVCKPMGSFKFSSSHAIAGIAFTWVMAMSCAAPPLFGWSRYIP
AncCyprin2(24) GEVALWSLVVLAIERYIVVCKPMGSFKFSSSHAFAGIAFTWVMAMACAAPPLVGWSRYIP
          (37) GEVALWSLVVLAIERYIVVCKPMGSFKFSSSHAFAGIAFTWVMAMACAAPPLVGWSRYIP
AncEutelo1(24) GQVALWSLVVLAIERYIVVCKPMGSFKFTATHAAAGVAFTWIMAMSCAAPPLFGWSRYIP
          (37) GQVALWSLVVLAIERYIVVCKPMGSFKFGASHAAAGVAFTWIMAMSCAAPPLFGWSRYIP
AncEutelo2(24) GQVALWSLVVLAIERYIVVCKPMGSFKFTATHAAAGVAFTWIMAMSCAAPPLFGWSRYIP
          (37) GQVALWSLVVLAVERYIVVCKPMGSFKFTATHAAAGVAFTWIMAMSCAAPPLFGWSRYIP
AncEutelo3(24) GQVSLWSLVVLAIERYIVVCKPMGSFKFTATHAAAGCAFTWIMAMSCAAPPLLGWSRYIP
          (37) GQVSLWSLVVLAVERYIVVCKPMGSFKFTATHAAAGCAFTWIMAMSCAAPPLLGWSRYIP
AncEutelo4(24) GEVALWSLVVLAVERYIVVCKPMGSFKFTGTHAAAGVLFTWIMAMACAAPPLFGWSRYIP
          (37) GEVALWSLVVLAVERYIVVCKPMGSFKFTGTHAAAGVLFTWIMAMACAAPPLFGWSRYIP
AncTetrapo(24) GQVALWSLVVLAIERYIVVCKPMGNFRFASTHAIMGIAFTWIMALACAAPPLFGWSRYIP
          (37) GQVALWSLVVLAIERYIVVCKPMGNFRFSSTHALMGIAFTWVMAFSCAAPPLFGWSRYIP
AncSaurops(24) GQVALWSLVVLAIERYIVVCKPMGNFRFSATHALMGIAFTWVMAFSCAAPPLFGWSRYIP
          (37) GQVALWSLVVLAIERYIVVCKPMGNFRFSATHALMGIAFTWVMAFSCAAPPLFGWSRYIP
AncSquamat(24) GQVALWSLVVLAIERYIVVCKPMGNFRFSATHALMGIAFTWFMAFSCAAPPLFGWSRYIP
          (37) GQVALWSLVVLAIERYIVVCKPMGNFRFSATHALMGIAFTWVMAFSCAAPPLFGWSRYIP

                    190       200       210       220       230       240
AncAgnatha(24) EGMQCSCGPDYYTLNPKYHNESYVIYMFVVHFLLPVTIIFFTYGRLICTVKEAAAQQQES
          (37) EGMQCSCGPDYYTLNPKYNNESYVIYMFVVHFVIPVTVIFFSYGRLICTVKEAAAQQQES
AncJawedFi(24) EGMQCSCGPDYYTLNPDYHNESYVMYMFVVHFLLPVTIIFFTYGRLICTVKEAAAQQQES
          (37) EGMQCSCGPDYYTLNPKYNNESYVIYMFVVHFVIPVTVIFFSYGRLICKVKEAAAQQQES
AncTeleost(24) EGMQCSCGPDYYTLNPDYHNESYVMYMFVVHFFLPVTIIFFTYGSLVCTVKAAAAQQQES
          (37) EGMQCSCGPDYYTLNPDYHNESYVIYMFVVHFFIPVTIIFFTYGSLVCTVKAAAAQQQES
AncClupeoc(24) EGMQCSCGPDYYTLNPDYNNESYVMYMFVCHFCLPVTIIFFTYGSLVCTVKAAAAQQQES
          (37) EGMQCSCGPDYYTLNPEYNNESYVIYMFVCHFCIPVTIIFFTYGSLVCTVKAAAAQQQES
AncCyprin1(24) EGMQCSCGPDYYTLNPEYNNESYVLYMFICHFCLPVTIIFFTYGSLVCTVKAAAAQQQES
          (37) EGMQCSCGPDYYTLNPEYNNESYVLYMFICHFCIPVTIIFFTYGSLVCTVKAAAAQQQES
AncCyprin2(24) EGMQCSCGPDYYTLNPEYNNESYVLYMFICHFILPVTIIFFTYGRLVCTVKAAAAQQQES
          (37) EGMQCSCGPDYYTLNPEYNNESYVLYMFICHFILPVTIIFFTYGRLVCTVKAAAAQQQES
AncEutelo1(24) EGMQCSCGPDYYTLNPGYNNESYVMYMFTCHFCLPVTIIFFTYGSLVCTVKAAAAQQQES
          (37) EGMQCSCGPDYYTLNPGYNNESYVMYMFTCHFCIPVTIIFFTYGSLVCTVKAAAAQQQES
AncEutelo2(24) EGMQCSCGPDYYTLAPGYNNESYVMYMFTCHFCVPVFIIFFTYGSLVMTVKAAAAQQQDS
          (37) EGMQCSCGPDYYTLAPGFNNESYVMYMFTCHFCVPVFIIFFTYGSLVMTVKAAAAQQQDS
AncEutelo3(24) EGIQVSCGPDYYTLAPGYNNESYVMYMFTCHFCVPVFTIFFTYGSLVMTVKAAAAQQQDS
          (37) EGIQVSCGPDYYTLAPGFNNESYVMYMFTCHFCVPVFTIFFTYGSLVMTVKAAAAQQQDS
AncEutelo4(24) EGMQCSCGPDYYTLAPGFNNESYVIYMFVVHFFVPVFIIFFTYGSLVMTVKAAAAQQQDS
          (37) EGMQCSCGPDYYTLAPGFNNESYVIYMFVVHFFVPVFIIFFTYGSLVMTVKAAAAQQQDS
AncTetrapo(24) EGMQCSCGPDYYTLNPDYHNESYVMYMFVVHFLLPVTIIFFTYGRLICKVKEAAAQQQES
          (37) EGMQCSCGPDYYTLNPDYHNESYVIYMFVVHFVIPVTVIFFSYGRLICKVKEAAAQQQES
AncSaurops(24) EGMQCSCGPDYYTLNPDYHNESYVLYMFVVHFIIPVVVIFFSYGRLICKVREAAAQQQES
          (37) EGMQCSCGPDYYTLNPDYHNESYVVYMFVVHFVIPVVVIFFSYGRLICKVREAAAQQQES
AncSquamat(24) EGMQCSCGPDYYTLNPDYHNESYVLYMFVVHFIIPVVVIFFSYGRLICKVREAAAQQQES
          (37) EGMQCSCGPDYYTLNPDYHNESYVVYMFVVHFVIPVVVIFFSYGRLICKVREAAAQQQES
```

(Fig. S1B, continued)

```
                          250       260       270       280       290       300
AncAgnatha(24)  ASTQKAEKEVTRMVIIMVIGFLVCWVPYASVAFYIFTNKGADFSATLMTVPAFFSKSSAL
          (37)  ASTQKAEKEVTRMVIIMVIGFLVCWVPYASVAFYIFTNKGSDFSATLMTVPAFFSKSSAL
AncJawedFi(24)  ASTQKAEKEVTRMVILMVIGFLVAWVPYASVAFWIFTNKGADFTATLMTVPAFFSKSSAL
          (37)  ASTQKAEKEVTRMVILMVIGFLVAWVPYASVAFWIFTNKGADFSATLMTVPAFFSKSSAL
AncTeleost(24)  ASTQKAEKEVTRMVILMVLGFLVAWTPYASVAAWIFFNKGAAFTAQSMAVPAFFSKSSAL
          (37)  ASTQKAEKEVTRMVILMVLGFLVAWTPYASVAAWIFFNKGAAFSAQSMAVPAFFSKSSAL
AncClupeoc(24)  ASTQKAEREVTRMVILMVLGFLVAWTPYASFAAWIFFNKGAAFTAQSMAVPAFFSKSSAL
          (37)  ASTQKAEREVTRMVILMVLGFLVAWTPYASFAAWIFFNKGAAFSAQSMAVPAFFSKSSAL
AncCyprin1(24)  ESTQKAEREVTRMVILMVLGFLVAWTPYASFAAWIFFNKGAAFSAQSMAVPAFFSKSSAL
          (37)  ESTQKAEREVTRMVILMVLGFLVAWTPYASFAAWIFFNKGAAFSAQSMAVPAFFSKSSAL
AncCyprin2(24)  ESTQKAEREVTRMVILMVLGFLVAWTPYATVAAWIFFNKGAAFSAQFMAVPAFFSKSSAL
          (37)  ESTQKAEREVTRMVILMVLGFLVAWTPYATVAAWIFFNKGAAFSAQFMAVPAFFSKSSAL
AncEutelo1(24)  ASTQKAEREVTRMCILMVLGFLVAWTPYASFAAWIFFNKGAAFTAQSMAVPAFFSKSSAL
          (37)  ASTQKAEREVTRMCVLMVLGFLVAWTPYASFAAWIFFNKGAAFTAQSMAVPAFFSKSSAL
AncEutelo2(24)  ASTQKAEKEVTRMCILMVLGFLVAWTPYASFAAWIFFNKGAAFTAMSMAIPAFFSKSSAL
          (37)  ASTQKAEKEVTRMCVLMVLGFLVAWTPYASFAAWIFFNKGAAFTAMSMAIPAFFSKSSAL
AncEutelo3(24)  ASTQKAEKEVTRMCILMVLGFLLAWTPYASFAAWIFFNKGAAFTAMSMAIPSFFSKSSAL
          (37)  ASTQKAEKEVTRMCILMVLGFLLAWTPYASFAAWIFFNKGAAFTAMSMAIPSFFSKSSAL
AncEutelo4(24)  ASTQKAEKEVTRMCVLMVMGFLVAWTPYASFAGWIFLNKGAAFTALTAAIPAFFAKSSAL
          (37)  ASTQKAEKEVTRMCVLMVMGFLVAWTPYASFAGWIFLNKGAAFTALTAAIPAFFAKSSAL
AncTetrapo(24)  ASTQKAEKEVTRMVILMVIGFLVAWVPYASVAFWIFTNKGADFTATLMTVPAFFSKSSAL
          (37)  ASTQKAEKEVTRMVILMVIGFLVAWTPYASVAFWIFTNKGADFSATLMTVPAFFSKSSAL
AncSaurops(24)  ASTQKAEKEVTRMVILMVLGFLLAWTPYAVVAFWIFTNKGADFTATLMTVPAFFSKSSSL
          (37)  ASTQKAEKEVTRMVILMVLGFLLAWTPYAVVAFWIFTNKGADFSATLMSVPAFFSKSSSL
AncSquamat(24)  ASTQKAEKEVTRMVILMVLGFLLAWTPYAVVAFWIFTNKGADFSATLMTVPAFFSKSSSL
          (37)  ASTQKAEKEVTRMVILMVLGFLLAWTPYAVVAFWIFTNKGADFSATLMSVPAFFSKSSSL

                          310       320       330       340       350
AncAgnatha(24)  YNPIIYVLLNKQFRNCMITTICCGKNPFGDEDVSSSVSQSKTEVSSVSSSQVSPA
          (37)  YNPIIYVLMNKQFRNCMITTICCGKNPFGDEDVSSSVSQSKTEVSSVSSSQVSPA
AncActino (24)  YNPIIYVLLNKQFRNCMITTICCGKNPFGDEDVSSSVSQSKTEVSSVSSSQVSPA
          (37)  YNPIIYVLMNKQFRNCMITTICCGKNPFGDEDVSSSVSQSKTEVSSVSSSQVSPA
AncTeleost(24)  YNPIIYVLLNKQFRNCMITTICCGKNPFGDEDVSSSVSQSKTEVSSVSSSQVSPA
          (37)  YNPIIYVLLNKQFRNCMITTICCGKNPFGDEDVSSSVSQSKTEVSSVSSSQVSPA
AncClupeoc(24)  YNPIIYVLLNKQFRNCMITTICCGKNPFGDEDVSSSVSQSKTEVSSVSSSQVSPA
          (37)  YNPIIYVLLNKQFRNCMITTICCGKNPFGDEDVSSSVSQSKTEVSSVSSSQVSPA
AncCyprin1(24)  YNPIIYVLLNKQFRNCMITTICCGKNPFGDEDVSSSVSQSKTEVSSVSSSQVSPA
          (37)  YNPIIYVLLNKQFRNCMITTICCGKNPFGDEDVSSSVSQSKTEVSSVSSSQVSPA
AncCyprin2(24)  YNPVIYVLLNKQFRNCMITTICCGKNPFGDEDVSSSVSQSKTEVSSVSSSQVSPA
          (37)  YNPVIYVLLNKQFRNCMITTICCGKNPFGDEDVSSSVSQSKTEVSSVSSSQVSPA
AncEutelo1(24)  FNPIIYVLMNKQFRNCMITTICCGKNPFGDEDVSSSVSQSKTEVSSVSSSQVSPA
          (37)  FNPIIYVLMNKQFRNCMITTICCGKNPFGDEDVSSSVSQSKTEVSSVSSSQVSPA
AncEutelo2(24)  FNPIIYVLMNKQFRNCMITTICCGKNPFGDEDVSSSVSQSKTEVSSVSSSQVSPA
          (37)  FNPIIYVLMNKQFRNCMITTICCGKNPFGDEDVSSSVSQSKTEVSSVSSSQVSPA
AncEutelo3(24)  FNPIIYILMNKQFRNCMITTICCGKNPFGDEDVSSSVSQSKTEVSSVSSSQVSPA
          (37)  FNPIIYILMNKQFRNCMITTICCGKNPFGDEDVSSSVSQSKTEVSSVSSSQVSPA
AncEutelo4(24)  YNPVIYVLMNKQFRNCMITTICCGKNPFGDEDVSSSVSQSKTEVSSVSSSQVSPA
          (37)  YNPVIYVLMNKQFRNCMITTICCGKNPFGDEDVSSSVSQSKTEVSSVSSSQVSPA
AncTetrapo(24)  YNPIIYVLLNKQFRNCMITTICCGKNPFGDEDVSSSVSQSKTEVSSVSSSQVSPA
          (37)  YNPIIYVLMNKQFRNCMITTICCGKNPFGDEDVSSSVSQSKTEVSSVSSSQVSPA
AncSaurops(24)  YNPIIYVLMNKQFRNCMITTICCGKNPFGDEDVSSSVSQSKTEVSSVSSSQVSPA
          (37)  YNPIIYVLMNKQFRNCMITTICCGKNPFGDEDVSSSVSQSKTEVSSVSSSQVSPA
AncSquamat(24)  YNPIIYVLMNKQFRNCMITTICCGKNPFGDEDVSSSVSQSKTEVSSVSSSQVSPA
          (37)  YNPIIYVLMNKQFRNCMITTICCGKNPFGDEDVSSSVSQSKTEVSSVSSSQVSPA
```

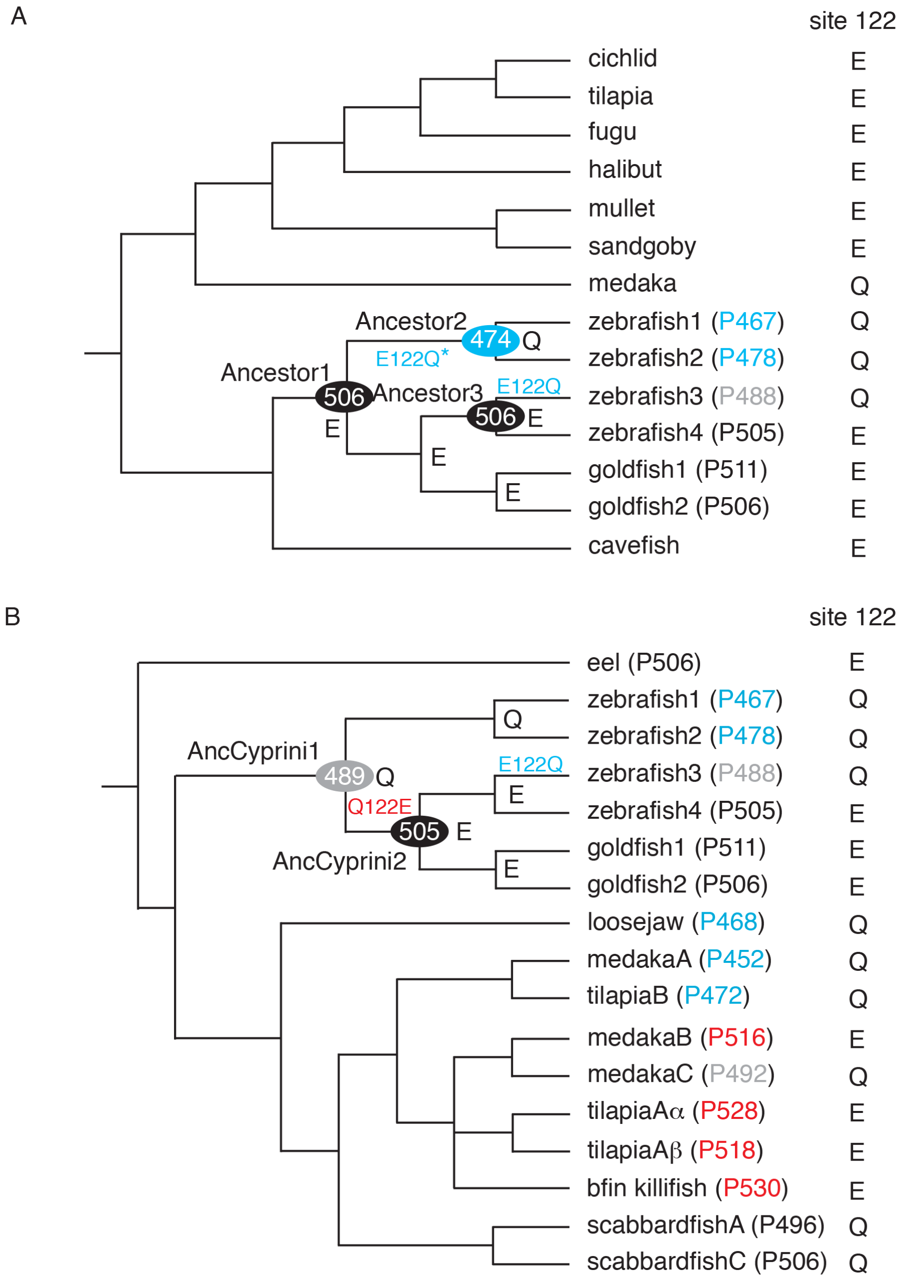

Fig. S2. Two different inferences of the RH2 pigment evolution in Cypriniformes. The AncCyprini1 was inferred to have either E122 (**A,** Chinen et al. 2005) or Q122 (**B**, present analysis). The λ_{max}s of Ancestors 1-3 are taken from (Chinen et al. 2005). The numbers in ovals and after P in rectangles show λ_{max}s of the ancestral and present-day pigments, respectively. The amino acids at site 122 are given at the right column. E122Q decreases the λ_{max}, whereas Q122E increases λ_{max}. E122Q* explains about 47% of the λ_{max}-shift of Ancestor2. Blue, grey, black, and red indicates the λ_{max}s of 452-478 nm, 488-492 nm, 495-511 nm, and 516-530 nm, respectively.

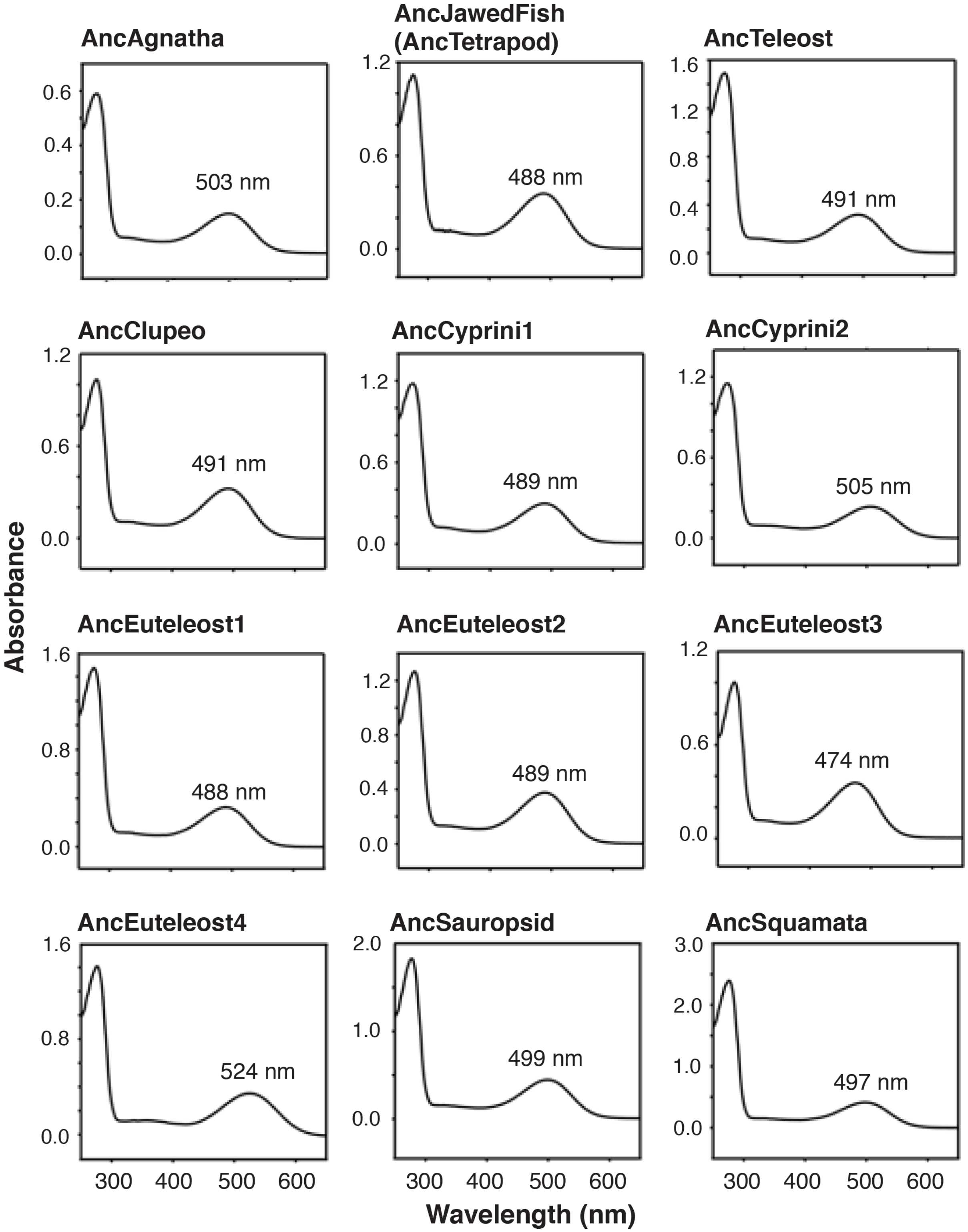

Fig. S3. Absorption spectra of ancestral RH2 pigments. The λ_{max} values of AncJawedFish and AncTetrapod are identical at 488 nm but their absorbance levels at ~280 nm are 1.1 and 1.6, respectively.
